# Synergy of nanodiamond–doxorubicin conjugates and PD-L1 blockade effectively turns tumor-associated macrophages against tumor cells

**DOI:** 10.1186/s12951-021-01017-w

**Published:** 2021-09-06

**Authors:** Hua-Zhen Xu, Tong-Fei Li, Chao Wang, Yan Ma, Yan Liu, Mei-Yan Zheng, Zhang-Jun-Yan Liu, Jin-Bo Chen, Ke Li, Shi-Kuan Sun, Naoki Komatsu, Yong-Hong Xu, Li Zhao, Xiao Chen

**Affiliations:** 1 Department of Pharmacology, School of Basic Medical Sciences, Wuhan University, Donghu Avenue No.185, Wuhan, 430072 China; 2 Department of Pharmacology, School of Basic Medical Sciences, Hubei University of Medicine, Hubei Key Laboratory of Embryonic Stem Cell Research, Taihe Hospital of Shiyan, Hubei University of Medicine, Renmin road No. 30, Shiyan, 442000 Hubei China; 3 Demonstration Center for Experimental Basic Medicine Education, School of Basic Medical Sciences, Wuhan University, Donghu Avenue No.185, Wuhan, 430072 China; 4 School of Material Science and Energy Engineering, Foshan University, Foshan, 528000 Guangdong China; 5 Graduate School of Human and Environmental Studies, Kyoto University, Sakyo-ku, Kyoto, 606-8501 Japan; 6 Institute of Ophthalmological Research, Department of Ophthalmology, Renmin Hospital of Wuhan University, Wuhan, 430060 China; 7 State Key Laboratory of Radiation Medicine and Protection, School of Radiation Medicine and Protection & School for Radiological and Interdisciplinary Sciences (RAD-X), Collaborative Innovation Center of Radiation Medicine of Jiangsu Higher Education Institutions, Soochow University, Suzhou, 215123 Jiangsu China; 8 Hubei Provincial Key Laboratory of Developmentally Originated Disease, Wuhan, 430071 China

**Keywords:** PD-L1/PD-1, Non-small cell lung cancer, Tumor-associated macrophages, HMGB1/RAGE/NF-κB signaling, Nanodiamond–doxorubicin conjugates

## Abstract

**Background:**

Tumor-associated macrophages (TAMs) are the most abundant stromal cells in the tumor microenvironment. Turning the TAMs against their host tumor cells is an intriguing therapeutic strategy particularly attractive for patients with immunologically “cold” tumors. This concept was mechanistically demonstrated on in vitro human and murine lung cancer cells and their corresponding TAM models through combinatorial use of nanodiamond-doxorubicin conjugates (Nano-DOX) and a PD-L1 blocking agent BMS-1. Nano-DOX are an agent previously proved to be able to stimulate tumor cells’ immunogenicity and thereby reactivate the TAMs into the anti-tumor M1 phenotype.

**Results:**

Nano-DOX were first shown to stimulate the tumor cells and the TAMs to release the cytokine HMGB1 which, regardless of its source, acted through the RAGE/NF-κB pathway to induce PD-L1 in the tumor cells and PD-L1/PD-1 in the TAMs. Interestingly, Nano-DOX also induced NF-κB-dependent RAGE expression in the tumor cells and thus reinforced HMGB1’s action thereon. Then, BMS-1 was shown to enhance Nano-DOX-stimulated M1-type activation of TAMs both by blocking Nano-DOX-induced PD-L1 in the TAMs and by blocking tumor cell PD-L1 ligation with TAM PD-1. The TAMs with enhanced M1-type repolarization both killed the tumor cells and suppressed their growth. BMS-1 could also potentiate Nano-DOX’s action to suppress tumor cell growth via blocking of Nano-DOX-induced PD-L1 therein. Finally, Nano-DOX and BMS-1 achieved synergistic therapeutic efficacy against in vivo tumor grafts in a TAM-dependent manner.

**Conclusions:**

PD-L1/PD-1 upregulation mediated by autocrine and paracrine activation of the HMGB1/RAGE/NF-κB signaling is a key response of lung cancer cells and their TAMs to stress, which can be induced by Nano-DOX. Blockade of Nano-DOX-induced PD-L1, both in the cancer cells and the TAMs, achieves enhanced activation of TAM-mediated anti-tumor response.

**Graphic abstract:**

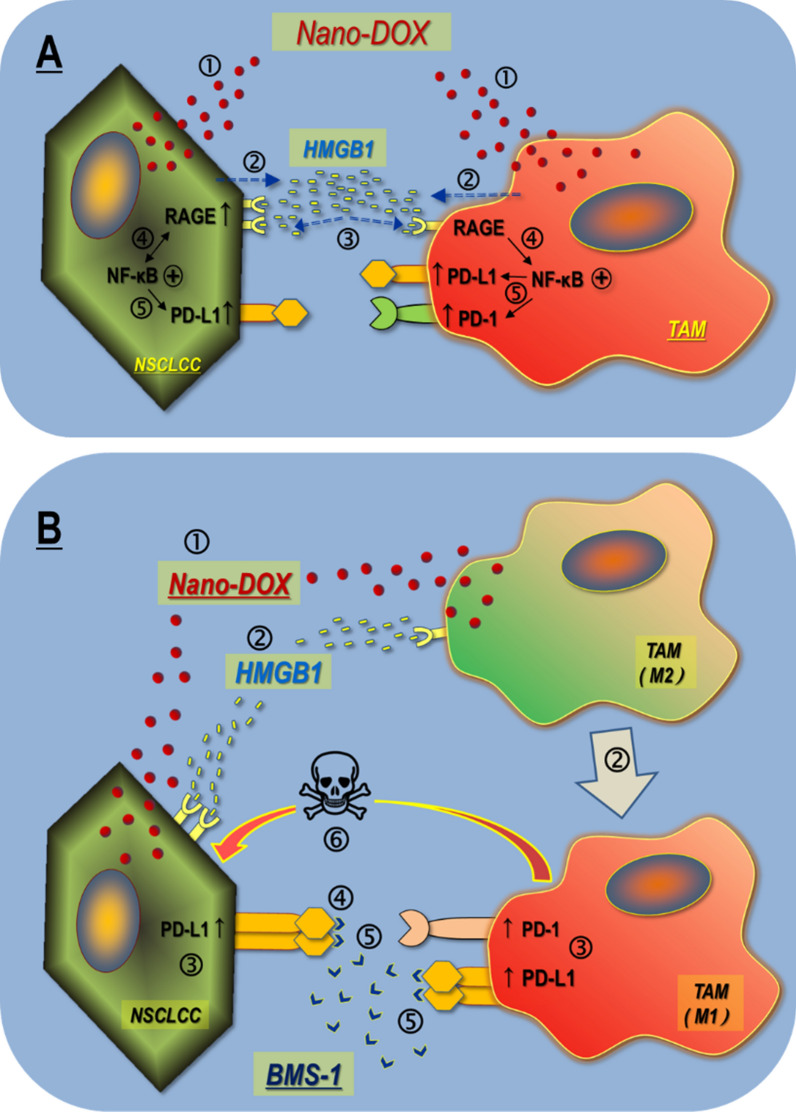

**Supplementary Information:**

The online version contains supplementary material available at 10.1186/s12951-021-01017-w.

## Introduction

Programmed death-ligand 1 (PD-L1) and its receptor, programmed cell death-1 (PD-1), are vital negative regulators of immune cell activation. PD-L1 is frequently expressed in many tumors to suppress anti-tumor immunity mediated by PD-1 positive tumor-infiltrating cytotoxic T lymphocytes through the PD-L1/PD-1 ligation [1]. Therapies that block PD-L1/PD-1 interaction between cancer cells and T cells thus promoting anti-tumor immunity have shown remarkable clinical efficacy in the treatment of a variety of malignant diseases [2–4]. However, these immunotherapies are largely ineffective in tumors with lymphocyte exhaustion or poor lymphocyte infiltration, a condition termed immunologically “cold” tumor [5–7]. Recently, PD-1 expression has been identified in some other immune components than the T cells in the tumor microenvironment (TME), particularly the tumor-associated macrophages (TAMs) [8, 9]. TAMs are the most abundant infiltrating leukocytes in the TME, accounting for up to 50% of the tumor mass in certain tumors, and have complex interactions with cancer cells [10–13]. It is intriguing to envision that blocking the PD-L1/PD-1 interaction between cancer cells and TAMs through anti-PD-L1/PD-1 therapy may turn the TAMs against their host cancer cells and thus achieve therapeutic efficacy, particularly in those immunologically cold tumors. The success of this strategy will hinge on the expression status of PD-L1/PD-1 in the tumor cells, the TAMs, and the activation phenotype of TAMs. There are complex interactions between tumor cells and their TAMs and the expression mechanisms of PD-L1/PD-1 in their interactions is poorly understood. Elucidation thereof not only holds uttermost importance in understanding the inner workings of the TME but also for the identification of targets critical for the development of novel and effective cancer therapies.

Nanotechnology has provided powerful tools for the modulation of the TME. We have previously fabricated nanodiamond-polyglycerol-doxorubicin conjugates (Nano-DOX), which is a delivery form of doxorubicin (DOX), and demonstrated that Nano-DOX, besides its tumor-suppressive action, could stimulate the immunogenicity of tumor cells and thereby elicit anti-tumor immune responses driven by TAMs and dendritic cells [14–16]. In addition to those already reported findings, there were some intriguing observations left unaccounted for from our previous work. The most outstanding thereof was that Nano-DOX could induce PD-L1 in cancer cells and PD-1 in the TAMs. As mentioned above, PD-L1 and PD-1 are negative regulators of immune cell activation, the upregulated PD-L1 and PD-1 are supposed to put a brake on the anti-tumor activation of TAMs induced by Nano-DOX. Thus, our immediate reaction to this observation was the vision that concurrent blockade of PD-L1/PD-1 should take the brake off and thus enhance the Nano-DOX-induced anti-tumor activation of TAMs, ultimately resulting in synergistic therapeutic efficacy. Before putting the idea to test, we decided in the first place to take a deeper look to identify the mechanisms of the PD-L1/PD-1 induction. Serving as a valuable clue as to where to begin the mechanistic exploration, another interesting observation caught our attention, which was that Nano-DOX also stimulated cancer cells to release high mobility group box 1 (HMGB1). HMGB1 is an architectural chromatin-binding protein that regulates nuclear homeostasis and genome stability [17]. Upon cell stress or injury, HMGB1 can be released to the outside of the cell as a member of the damage-associated molecular patterns (DAMPs) and functions as a pro-inflammatory cytokine that can activate macrophages through binding with receptors including the receptor for advanced glycation endproducts (RAGE), Toll-like receptors (TLR2, TLR4, and TLR9) and CXCR4 [18, 19]. Recently, melanoma cells subjected to ultraviolet radiation (UVR) were shown to release HMGB1 which subsequently activated RAGE to promote nuclear factor-κB (NF-κB)-dependent transcription of PD-L1 in melanoma cells [20]. On the other hand, HMGB1 released by esophageal squamous cell carcinoma cells was found to induce PD-1^+^ TAM expansion [21]. These findings prompted us to hypothesize that Nano-DOX may stimulate cancer cells to release HMGB1 which induces PD-L1 in the cancer cells and PD-1 in the TAMs via activation of the RAGE/NF-κB signaling axis. To substantiate the hypothesis, we performed experiments on human and murine non-small cell lung cancer (NSCLC) and TAM models. Free DOX was also investigated wherever possible for comparison with Nano-DOX. HMGB1 release, PD-L1 induction in the cancer cells, and PD-1 induction in the TAMs were first examined. The activity of the HMGB1/RAGE/NF-κB pathway in the cancer cells and TAMs was then probed for the mechanism of PD-L1 induction and PD-1 induction. As it transpires, the obtained findings not only substantiate but also expand the notion of our original hypothesis. Of note, Nano-DOX was also found to act differently than DOX. Following the mechanistic study, the same in vitro cell models were used to demonstrate the synergy of Nano-DOX and BMS-1, a PD-L1 blocker, in terms of TAM reactivation and anti-tumor action. Our initial expectation was that PD-L1 blockade (by BMS-1) would enhance Nano-DOX’s anti-NSCLC action in a TAMs-dependent manner. But again, discoveries were made revealing more than expected. Finally, in vivo experiments were carried out on mice bearing NSCLC tumor grafts with or without TAM depletion to corroborate the in vitro findings and demonstrate the therapeutic synergy between Nano-DOX and BMS-1. Our findings are presented in this manuscript and the implications, significance, and biomaterial aspects thereof in tumor therapy are discussed.

## Materials and methods

### Nano-DOX and BMS-1

**Fig. 1:**
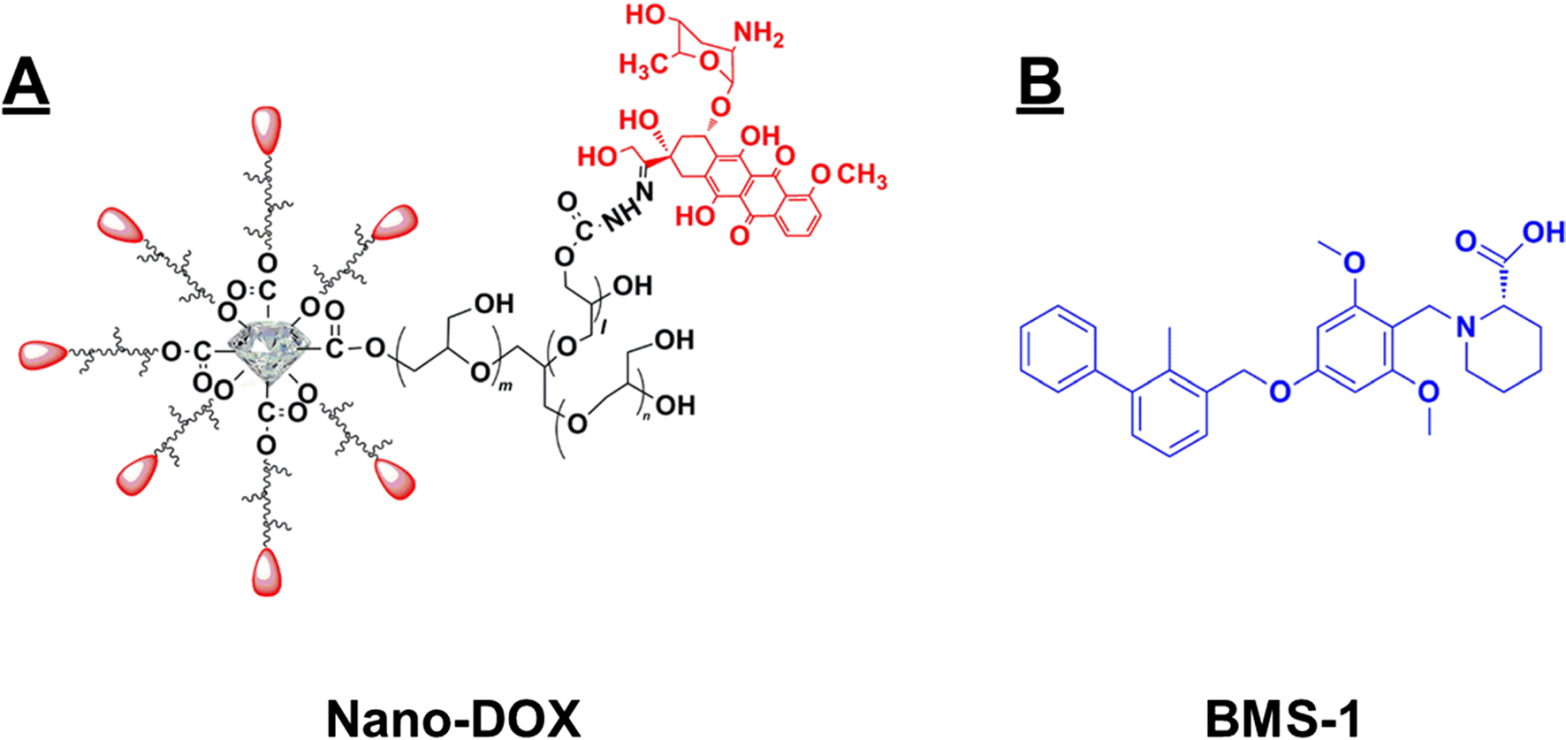
Molecular structure of Nano-DOX and BMS-1

This device was developed based on nanodiamonds (4–5 nm in diameter) with surface functionalization of polyglycerol (Nd-PG). DOX was loaded to the Nd-PG giving Nano-DOX. Nano-DOX has an aqueous hydrodynamic diameter of 83.9 ± 32.3 nm and has good solubility in physiological solutions. The synthesis and characterization of Nano-DOX were detailed in a previously published paper [22]. Figure 1 shows the structural composition and size of Nano-DOX. The Nano-DOX stock solution in water was kept at 4 °C and was sonicated in a water bath for 3 min before being diluted with culture medium into working concentrations. All concentrations and dosages of Nano-DOX were normalized to DOX. BMS-1 is one of a series of small molecule agents that can induce PD-L1 dimerization and thereby blocks its interaction with PD-1 [23–25].

### Cell models

Mouse and human NSCLC models, the Lewis and A549 NSCLC cells, were obtained from the Cell Bank of Shanghai Institutes for Biological Sciences (Shanghai, China). The human TAM model with the type-2 phenotype (hM2) was differentiated from THP-1 cells, a monocytic leukemia cell line, according to a previously published protocol [14]. The mouse TAM model with the type-2 phenotype (mM2) was isolated and differentiated from mouse bone marrow according to published protocols [14, 26]. All cells were cultured in RPMI-1640 medium (Sigma-Aldrich, USA) supplemented with 10% fetal bovine serum (Sigma-Aldrich) in a humidified incubator (5% CO_2_/95% air atmosphere at 37 °C).

### DAMPs emission

A549 or Lewis cells in 24-well plates with a seeding density of 2.5 × 10^5^ cells/well were treated with either Nano-DOX or DOX at 2 μg/mL for 24 h. Cell surface CRT and HSP90 were detected by immunofluorescent staining and flow cytometry (FACS). Culture medium supernatants were collected and HMGB1 levels were determined by ELISA (Elabscience, E-EL-H1554c) with a Biotek ELX800 microplate reader, and ATP levels were determined with a Chemiluminescence ATP Determination Kit (Beyotime, S0027, China) and an illuminometer (Tecan, Spark 10 M).

### PD‑1/PD-L1 expression

A549 or Lewis cells (2 × 10^5^ cells/well) labeled with carboxyfluorescein succinimidyl ester (CFSE) [14] either in a single culture or mixed-cultured with the TAMs (hM2 or mM2) (2 × 10^5^ cells/well) in 24-well plates were treated with Nano-DOX (2 μg/mL) for 24 h. The cells were then collected and immunofluorescent staining and FACS were performed to assay cell surface PD-L1/PD-1 abundance.

### Blocking of the HMGB1/RAGE/NF-κB pathway

Cells in 24-well plates were pretreated with 20 µM of glycyrrhizic acid (GA), 2 mM of ethyl pyruvate (EP), 2 µM of FPS-ZM1, and 2 µM of pyrrolidine dithiocarbamate (PDTC) for 2 h, respectively. Recombinant HMGB1 (rHMGB1) (10,326-H08H Sino Biological), 0.5 µg/mL for the hM2 and 2 µg/mL for the A549 cells [27–29], or Nano-DOX (2 µg/mL) was then added to the cells. After 24-h incubation, the cells were collected and immunofluorescent staining and FACS, and western blotting were performed to assay PD-L1 or PD-1 expression.

### TAM phenotyping

The hM2 or mM2 cells in 24-well plates (2 × 10^5^ cells/well), either in a single culture or mixed-cultured with CFSE-labeled A549 or Lewis cells (2 × 10^5^ cells/well) in 24-well plates, were treated with Nano-DOX (2 μg/mL), BMS-1 (1 µM), or Nano-DOX plus BMS-1 for 24 h. The cells were then taken and immunofluorescent staining of cell surface CD80, CD86, MHC-II, and phagocytosis function were analyzed by FACS. The hM2 or mM2 cells in 6-well plates (1.2 × 10^6^ cells/well) were treated with Nano-DOX or BMS-1, or Nano-DOX plus BMS-1 for 24 h. The cells were then taken and GBP5 protein levels were assayed by western blot.

### Phagocytosis assay

Macrophage phagocytic function was assayed using fluorescent latex beads (2 μm, blue, Sigma L0280). The beads were re-suspended in PBS supplemented with 50% FBS, and subsequently added to cells and incubated at 37 °C for 2 h. Cells were then washed with pre-cooled PBS and analyzed via FACS [30].

### Cell proliferation and apoptosis

CFSE-labeled A549 or Lewis cells (2 × 10^5^ cells/well) in a single culture or in mixed culture with hM2 or mM2 (2 × 10^5^ cells/well) in 24-well plates were treated with Nano-DOX or BMS-1, or Nano-DOX plus BMS-1 for 24 h. The cells were then taken and cell surface annexin v staining was measured by FACS and decay of CFSE staining indicative of cell proliferation was analyzed by FACS per a previously published protocol [15].

### Immunofluorescent staining and fluorescent microscopy

Cells were fixed with paraformaldehyde (4%) and then blocked with 5% BSA in 1 × PBS at 37 °C for 1 h. Cells were then incubated with primary antibodies against CRT, BAX, and NF-κB at 4 °C overnight. The stained cells were washed 3 times with PBST (1% Tween-20 in 1 × PBS), incubated with Alexa Fluor 647-conjugated secondary antibody (bs-0295G, Bioss) at 37 °C for 2 h, and then washed 3 times with 1 × PBS. Finally, the cells were stained with Hoechst 33342 (5 μg/mL) for 15 min at room temperature and washed 3 times with 1 × PBS. Samples were then examined under a confocal microscope (Leica-LCS-SP8-STED, Germany).

### Western blotting

Cells subjected to required treatments in six-well plates were rinsed twice with ice-cold PBS and lysed in RIPA buffer with a 1% protease inhibitor cocktail. Cell lysates were cleared by centrifugation and protein concentration was determined using a BCA kit. Equal amounts of proteins were fractionated by SDS-PAGE and transferred to a PVDF membrane. The membranes were blocked with 5% fat-free milk in TBST and incubated with antibodies against PCNA, Ki67, NF-κB, Phospho- NF-κB, PD-L1, PD1, GBP5, β-actin, and GADPH overnight at 4 °C. Protein bands were imaged using a horseradish peroxidase-conjugated secondary antibody and ECL and the films were exposed using a Bio Imaging system (Syngene).

### FACS assay

FACS was performed using a flow cytometer (BD, FACS AriaIII, USA). Antibody fluorescent staining of CRT, HSP90, PD-L1, PD1, RAGE, CD80, CD86, MHC-II, and CFSE fluorescence were acquired in the FITC channel. DOX and Nano-DOX fluorescence was acquired in the PE channel. At least 10,000 events were collected per sample. Geometric means (GM) were used to quantify the fluorescent intensity.

### Mouse NSCLC homografts and treatments

Female athymic BALB/c nude mice at 4–5 weeks of age (18–20 g) were purchased from Shanghai Laboratory Animal Center at the Chinese Academy of Sciences (Shanghai, China). Animal handling and experimental procedures were in line with protocols approved by the Animal Care Committee at the Wuhan University. Mice were housed in a temperature-controlled environment with fresh water and a rodent diet available at all times. All inoculations and administrations were performed under Nembutal anesthesia. For the establishment of tumor homografts, each mouse was subcutaneous injected at the left armpit with Lewis cells (3 × 10^6^ cells/200 μL in PBS). The animals were randomly grouped into eight groups. (4 mice per group). Four groups were administered liposome chlorophosphate (LIPOSOMA) to deplete macrophages and the rest were treated with empty liposomes. The LIPOSOMA (5 mg/mL, 200 μL per mouse, i.v.) was administrated 24 h after Lewis cell inoculation, three times a week for one month [31]. When the tumor volume reached 100–400 mm^3^, Nano-DOX (4 mg/kg, i.v.), BMS-1 (2.5 mg/kg, i.p.), and Nano-DOX plus BMS-1 were given once every other day for 3 weeks, respectively. Animals in group “Control” only received PBS. Animal body weight and tumor size were taken every day. All animals were sacrificed at the end of the treatment duration and vital organs were harvested and weighed. Cryosections (5 μm) of tumor tissues were prepared for fluorescent microscopy and paraffin sections were prepared for immunohistochemical (IHC) staining. The efficiency of macrophage depletion was assessed by IHC analysis of macrophage surface marker CD11b (Additional file 1: Figure S10). Tumor growth curves over the treatment duration were obtained by plotting the tumor volume taken every other day versus time. Growth rates over the treatment duration (from day 13 to day 27) were calculated by regression analysis.

### IHC analysis

Antibodies for IHC analysis included CRT (ab92516, Abcam), HSP90 (ab13495, Abcam), HMGB1 (ab79823, Abcam), PD-L1 (17952-1-AP, Proteintech), PD1 (18106-1-AP, Proteintech), CD11b (20991-1-AP, Proteintech), CD206(ab64693, Abcam), CD80 (BS-2211R,BIOSS), CD86 (ab213044, Abcam), MHCII (sc-59318, Santa Cruz), KI67 (ab16667, Abcam), PCNA (ab92552, Abcam), BAX (50599-2-lg, Proteintech), CASPASE3 (19677-1-AP,Proteintech), RAGE (bs-0177R) GBP5 (132201-AP, Proteintech), NF-κB (10745–1-AP, Proteintech), Phospho- NF-κB (bs-0982R, Bioss). Paraffin sections (5 μm) were dewaxed and rehydrated, antigen repaired with sodium citrate for 20 min, then incubated in 3% hydrogen peroxide for 10 min at room temperature. The paraffin sections were then blocked with 5% BSA for 30 min, stained with antibodies overnight at 4 °C, washed with PBS, and stained with secondary antibody (PV-9000, ZSGB-BIO) for 1 h at 37 °C. DAB (ZLI-9018, ZSGB-BIO) was applied for coloration for 5 min at room temperature. Hematoxylin was used to stain the nucleus.

### Statistical analysis

Quantitative data are expressed as means ± standard deviation (SD) and subjected to One-way analysis of variance (ANOVA) to determine if there are any statistically significant differences between the treatment groups.

## Results

### Nano-DOX induced PD-L1 in the NSCLC cells and PD-1 in the TAMs via activation of the HMGB1/RAGE/NF-κB pathway

#### Nano-DOX induced cancer cell emission of HMGB1 and other DAMPs

**Fig. 2:**
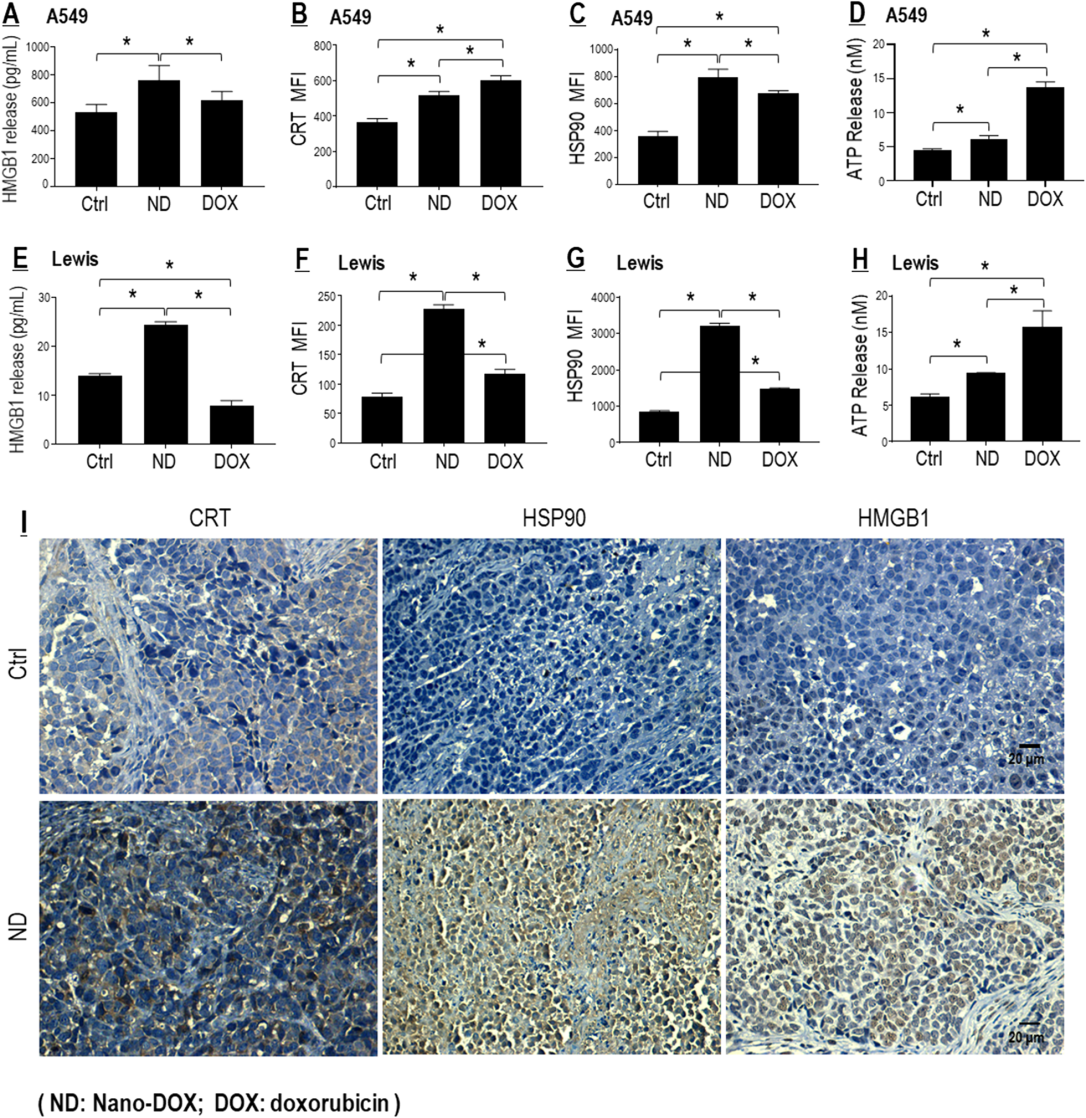
Nano-DOX stimulated DAMPs emission from NSCLC cells. **A**–**H** Nano-DOX stimulated emission of HMGB1, CRT, HSP90 and ATP in in vitro A549 and Lewis cells. HMGB1 was assayed by ELISA. CRT and HSP90 was assayed by FACS analysis of immunofluorescent staining, and ATP assayed with a bioluminescence assay kit. FACS histogram geometric means were used to quantify mean fluorescence intensity (MFI). Values are means ± SD (*n* = 3, *p < *0.05*). **I** Nano-DOX treatment resulted in increased immunohistological staining of DAMPs (CRT, HSP90 and HMGB1) in subcutaneous xenografts of Lewis cells in mice. Drug concentration was 2 μg/mL for DOX and Nano-DOX in the in vitro experiments and treatment duration was 24 h. Representative FACS dot plots for **B**, **C**, **F** and **G** were provided in Additional file 1: Figure S2

DAMPs are endogenous adjuvant molecules released by damaged or dying cells, which can initiate inflammation and stimulate the innate immune response. Thus, DAMPs release is indicative of cell injury and increased immunogenicity. Our previous work had demonstrated that Nano-DOX could stimulate glioblastoma cells to release DAMPs including HMGB1, adenosine triphosphate (ATP), heat shock protein 90 (HSP90), and calreticulin (CRT) [14]. The same effect was observed in the NSCLC cells (i.e. A549 and Lewis), both in vitro and in vivo (Fig. 2). The DAMPs releasing action of Nano-DOX was generally along the line of DOX, only with different potency. One exception was HMGB1, whose emission was reduced by DOX. In agreement with their capacity to stimulate DAMPs release, both DOX and Nano-DOX impaired the viability of NSCLC cells, with higher potency seen with DOX (Additional file 1: Figure S1 A, B).

#### Nano-DOX induced PD-L1 in the NSCLC cells and PD-1 in the TAMs

**Fig. 3:**
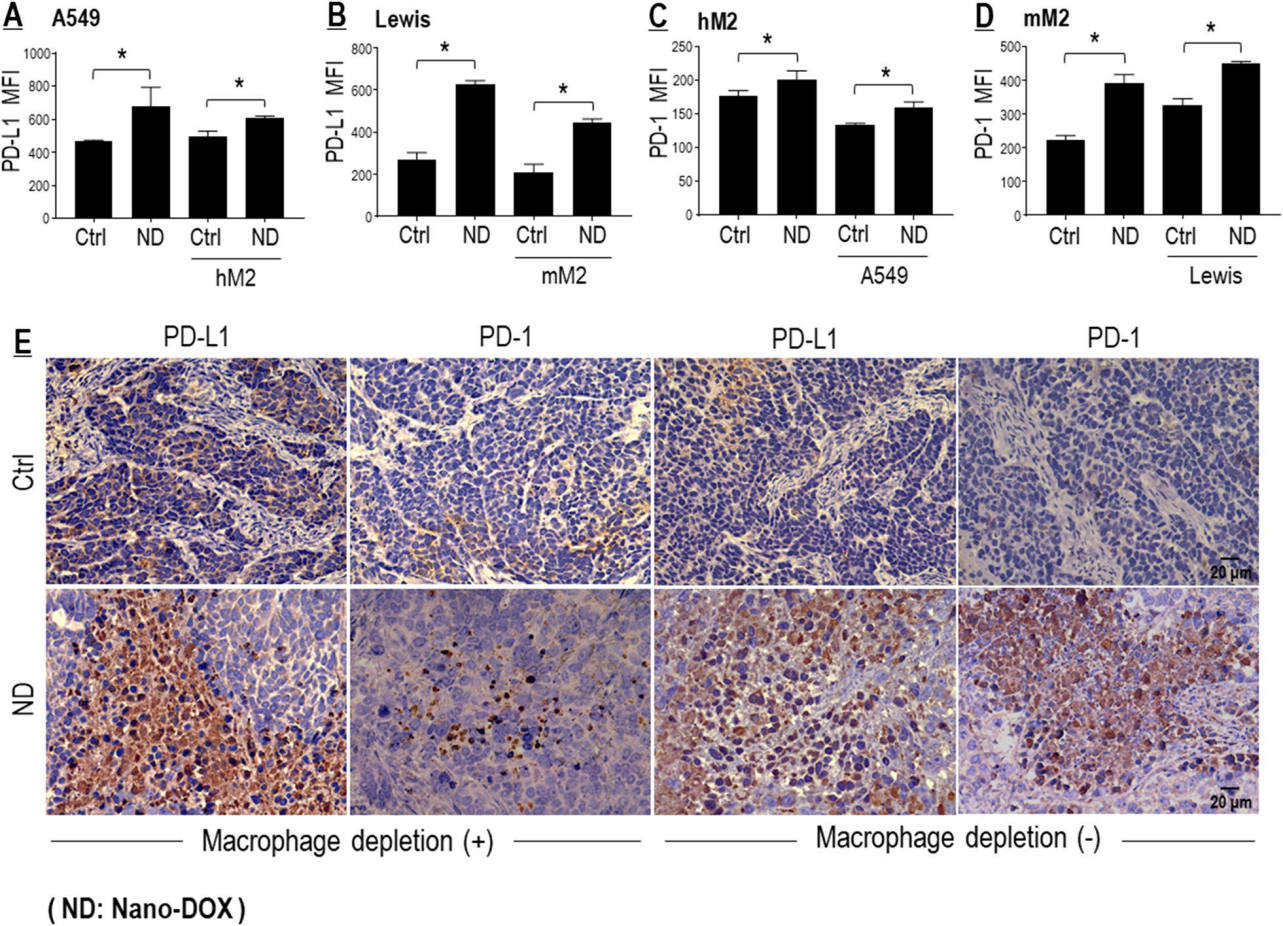
Nano-DOX induced PD-L1 in NSCLC cells and PD-1 in TAMs. **A**–**D** Nano-DOX induced PD-L1 in the in vitro cancer cells (A549 & Lewis) and PD-1 in the in vitro TAM models (hM2 & mM2) in monoculture and mixed-culture. Cell surface PD-L1 and PD-1 were assayed by FACS analysis of immunofluorescent staining. **E** Nano-DOX treatment led to increased immunohistological staining of PD-L1 and PD-1 in subcutaneous xenografts of Lewis cells in mice. FACS histogram geometric means were used to quantify mean fluorescence intensity (MFI). Geometric means were used to quantify mean fluorescence intensity (MFI). Values were means ± SD (*n* = 3, *p < *0.05*). Drug concentration was 2 μg/mL for DOX and Nano-DOX in the in vitro experiments and treatment duration was 24 h. Representative FACS dot plots for **A**–**D** were provided in Additional file 1: Figure S3

Next, we confirmed that Nano-DOX could induce PD-L1 in the NSCLC cells and PD-1 in the TAMs, both in the single and mixed culture (Fig. 3A–D). The TAM models were type II-activated macrophages derived from human THP-1 cells (hM2) and mouse bone marrow (mM2). Notably, induction of PD-L1 and PD-1 by Nano-DOX was more dramatic in the mouse NSCLC and TAMs models (Lewis & mM2) than their human counterparts (A549 & hM2). Induction of PD-L1 and PD-1 was also detected in in-vivo Lewis tumor grafts treated with Nano-DOX (Fig. 3E). PD-1 induction in tumor grafts was much lesser when TAMs had been depleted (Fig. 3E). DOX was also found to induce PD-L1 in the NSCLC cells (Additional file 1: Figure S3E, F). But DOX was not compared with Nano-DOX on the TAM models due to their intolerance of DOX. As shown in Additional file 1: Figure S1 C & D, the TAM models were very sensitive to DOX’s toxicity but tolerated Nano-DOX well. These observations are in keeping with our previous findings [14, 15, 22].

#### Nano-DOX induced PD-L1 in NSCLC cells through reinforced activation of the HMGB1/RAGE/NF-κB pathway

**Fig. 4:**
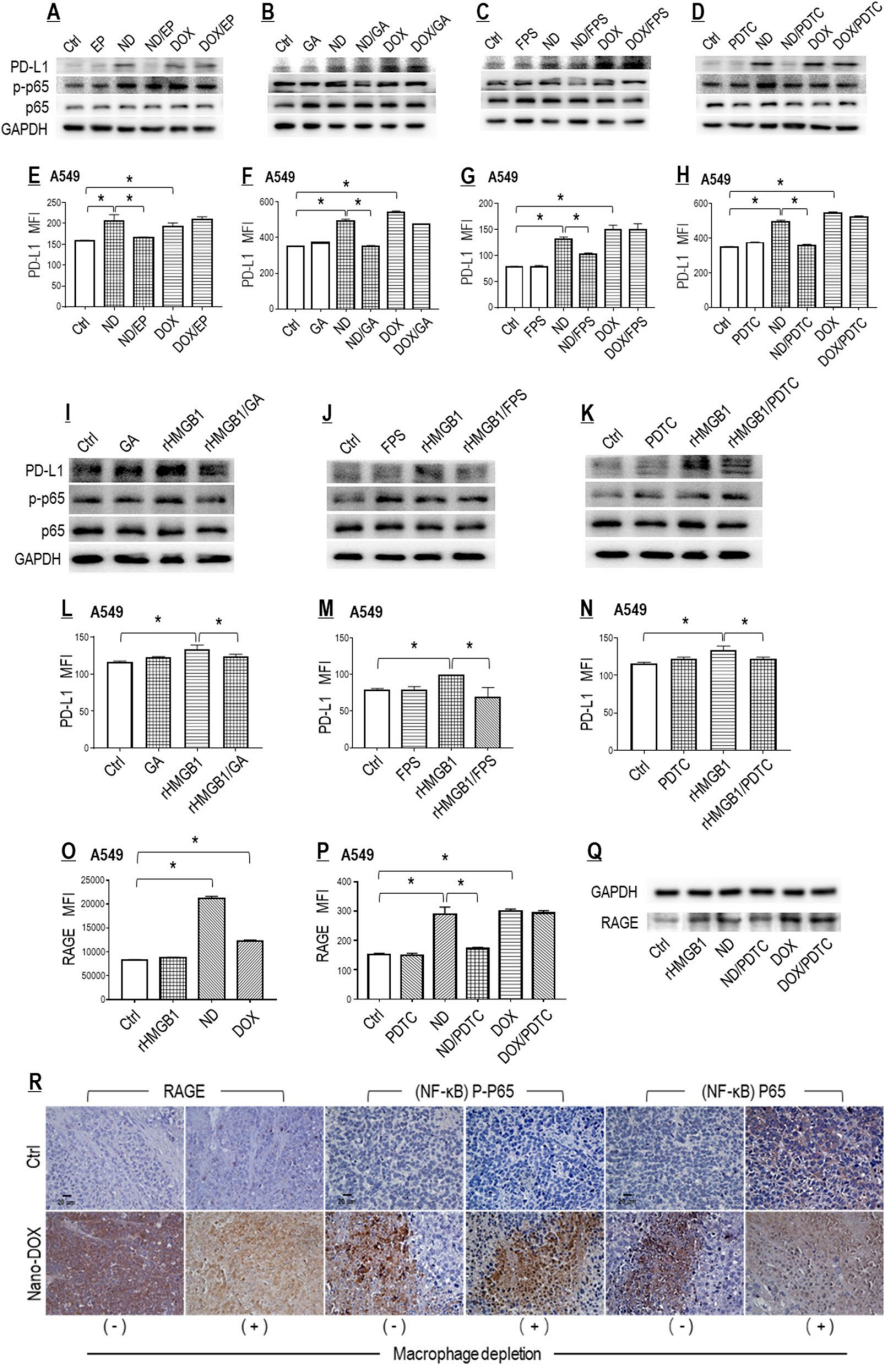
Nano-DOX induced PD-L1 in NSCLC cells through reinforced activation of the HMGB1/RAGE/NF-κB pathway. **A**–**H** Pharmacological blocking of the HMGB1/RAGE/NF-κB pathway suppressed PD-L1 induction by Nano-DOX but not DOX in the in vitro A549 cells. **I**–**N** HMGB1 induced PD-L1 in the in vitro A549 cells, which was repressed by blocking of the HMGB1/RAGE/NF-κB pathway. **O** Nano-DOX and DOX increased cell surface RAGE. Exogenously given HMGB1 was negative of this effect. **P**, **Q** Blocking of NF-κB repressed PD-L1 induction by Nano-DOX but not DOX. Exogenously given HMGB1 also increased protein level of RAGE. Cell surface PD-L1 and RAGE were assayed by FACS analysis of immunofluorescent staining and protein levels thereof were assayed by western blotting. **R** Nano-DOX treatment led to increased immunohistological staining of RAGE and activated NF-κB in subcutaneous xenografts of Lewis cells in mice. FACS histogram geometric means were used to quantify mean fluorescence intensity (MFI). Values were means ± SD (*n* = 3, *p < *0.05*). EP is an inhibitor of HMGB1 secretion. GA both neutralizes HMGB1’s cytokine activity and suppresses its secretion. FPS-ZM1 is a high-affinity inhibitor of RAGE. PDTC is a selective inhibitor of NF-κB. Drug concentration was 2 μg/mL for DOX and Nano-DOX in the in vitro experiments and treatment duration was 24 h. Representative FACS dot plots for **E**–**H** and **L**–**P** were provided in Additional file 1: Figure S4. Effect of EP alone on surface PD-L1 expression in A549 cells is shown in Additional file 1: Figure S4 E’

To establish the autocrine HMGB1/RAGE/NF-κB pathway as the causal link between Nano-DOX treatment and PD-L1 induction in the NSCLC cells, the A549 cells were treated with Nano-DOX or HMGB1 while one component of the pathway (i.e. HMGB1, RAGE, or NF-κB) was pharmacologically blocked, before total protein level and cell surface abundance of PD-L1 were examined. Ethyl pyruvate (EP) is an inhibitor of HMGB1 secretion [32]. Glycyrrhizic acid (GA) both neutralizes HMGB1’s cytokine activity and suppresses its secretion [33]. FPS-ZM1 is a high-affinity inhibitor of RAGE [34]. Pyrrolidine dithiocarbamate (PDTC) is a selective inhibitor of NF-κB [35]. As shown in Fig. 4A–H, each of these agents invariably blocked Nano-DOX-induced PD-L1 expression as well as NF-κB activation (phosphorylation). Consistently, direct exposure of recombinant HMGB1 (rHMGB1) also raised PD-L1 expression, and this effect was alleviated by GA, FPS-ZM1, and PDTC, respectively (Fig. 4I–N). But PD-L1 induction by Nano-DOX appeared more dramatic than by HMGB1. We postulated that the expression status of RAGE, the receptor that mediates HMGB1’s effect, might play some role in this difference. Intriguingly, Nano-DOX was indeed found to stimulate RAGE expression, both in total protein and in cell surface abundance, in an NF-κB-dependent manner whereas HMGB1 only increased the total protein of RAGE (Fig. 4O–Q). In-vivo Lewis tumor grafts treated with Nano-DOX also displayed RAGE upregulation and NF-κB activation (Fig. 4R). Taken together, these observations strongly suggest that Nano-DOX promote NF-κB-dependent PD-L1 expression in the NSCLC cells via enhanced activation of autocrine HMGB1-RAGE interaction by stimulating HMGB1 secretion and RAGE expression at the same time. An important deduction herein is that Nano-DOX, by virtue of their RAGE-induction property, may also potentiate tumor cell RAGE interaction with HMGB1 derived from tumor stromal cells, e.g. the TAMs.

Importantly, DOX also induced PD-L1 expression in the A549 cells but probably not via the HMGB1/RAGE/NF-κB pathway as none of the pathway’s blockers could suppress PD-L1 induction by DOX (Fig. 4A–H). DOX also induced RAGE expression, but independent of NF-κB (Fig. 4O–Q).

#### Nano-DOX induced PD-1 in TAMs through activation of the HMGB1/RAGE/NF-κB pathway

**Fig. 5:**
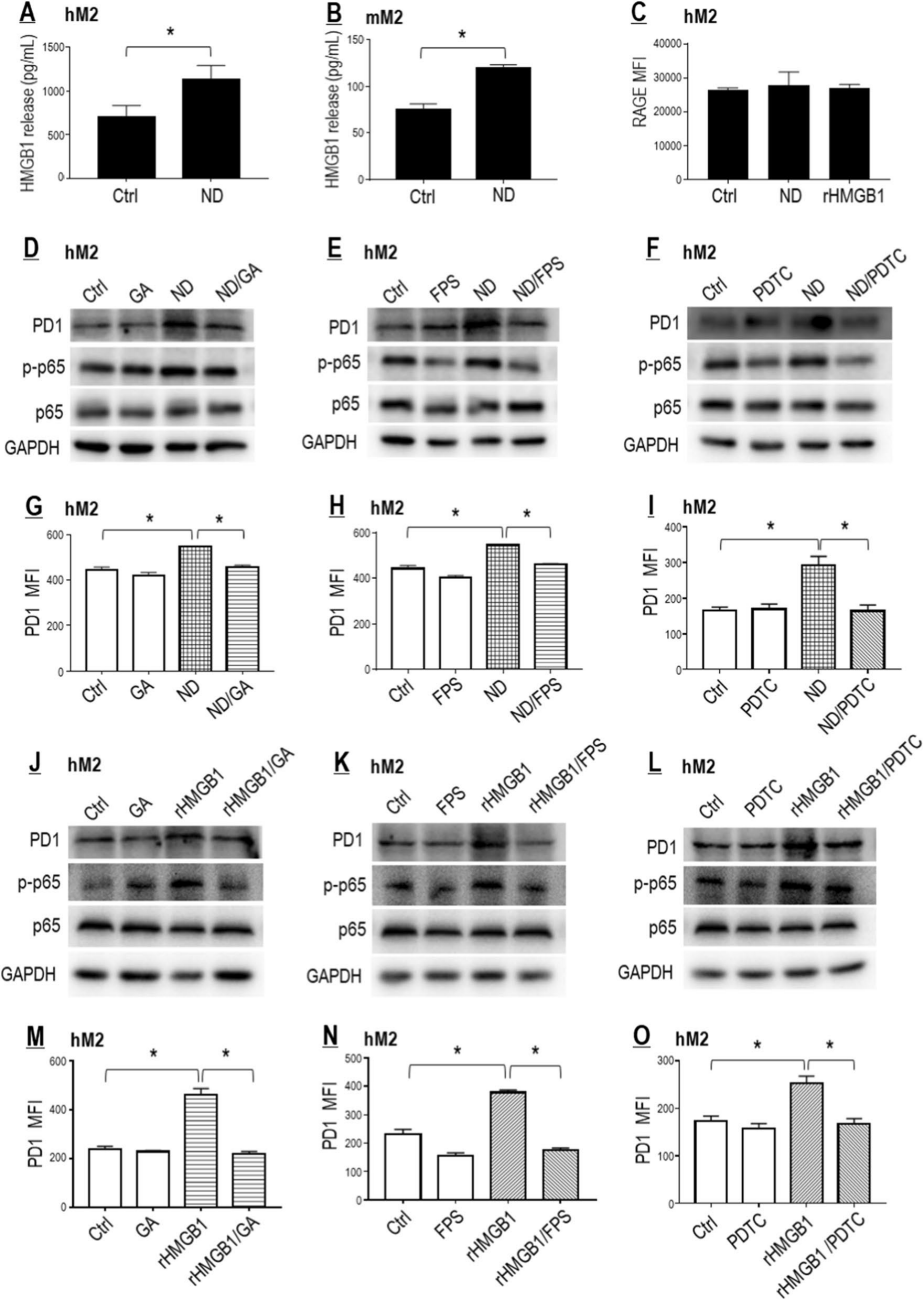
Nano-DOX induced PD-1 in TAMs through activation of the HMGB1/ RAGE/NF-κB pathway. **A**, **B** Nano-DOX stimulated HNGB1 secretion from the hM2 and mM2. **C** Neither Nano-DOX nor HMGB1 induced RAGE in the hM2. **D**–**I** Pharmacological blocking of the HMGB1/RAGE/NF-κB pathway suppressed PD-1 induction by Nano-DOX in the hM2. **J**–**O** HMGB1 induced PD-1 in the hM2, which was repressed by blocking of the HMGB1/RAGE/NF-κB pathway. Cell surface PD-1 and RAGE were assayed by FACS analysis of immunofluorescent staining and protein levels thereof were assayed by western blotting. FACS histogram geometric means were used to quantify mean fluorescence intensity (MFI). Values were means ± SD (*n* = 3, *p < *0.05*). Glycyrrhizic acid (GA) both neutralizes HMGB1’s cytokine activity and suppresses its secretion. FPS-ZM1 is a high-affinity inhibitor of RAGE. Pyrrolidine dithiocarbamate (PDTC) is a selective inhibitor of NF-κB. Drug concentration was 2 μg/mL for DOX and Nano-DOX and HMGB1 (0.5 μg/mL for hM2 and 2 μg/mL for the A549 cells) in the in vitro experiments and treatment duration was 24 h. Representative FACS dot plots for **C**, **G**–**I** and **M**–**O** were provided in Additional file 1: Figure S5

We initially only assumed tumor cell-derived HMGB1 to be the driving force of PD-1 induction in the TAMs. However, Nano-DOX was also found to stimulate HMGB1 secretion from the TAMs, albeit with no effect on RAGE expression (Fig. 5A–C). To explore the role of autocrine HMGB1/RAGE/NF-κB pathway between Nano-DOX treatment and PD-1 induction in the TAMs, the hM2 were treated with Nano-DOX or HMGB1 while one component of the pathway (i.e. HMGB1, RAGE, or NF-κB) was pharmacologically blocked, before total protein level and cell surface abundance of PD-1 were examined. Similar to Nano-DOX, exogenously given rHMGB1 markedly increased PD-1 expression in the TAMs and blocking the HMGB1/RAGE/NF-κB pathway by GA or FPS-ZM1 or PDTC repressed PD-1 upregulation induced either by Nano-DOX or HMGB1 (Fig. 5D–O). These observations indicate that Nano-DOX may activate the autocrine HMGB1/RAGE/NF-κB pathway to promote PD-1 expression in the TAMs. Importantly, as mentioned earlier, tumor cell-derived HMGB1 may also act on RAGE in the TAMs to promote NF-κB-dependent PD-1 expression given the spatial proximity between TAMs and their host tumor cells in the tumor tissues.

### PD-L1 blockade and Nano-DOX synergistically repolarized the TAMs into an M1-like phenotype

#### BMS-1 enhanced Nano-DOX-stimulated M1-type activation of TAMs both in a tumor cell-dependent and a tumor cell-independent manner

**Fig. 6:**
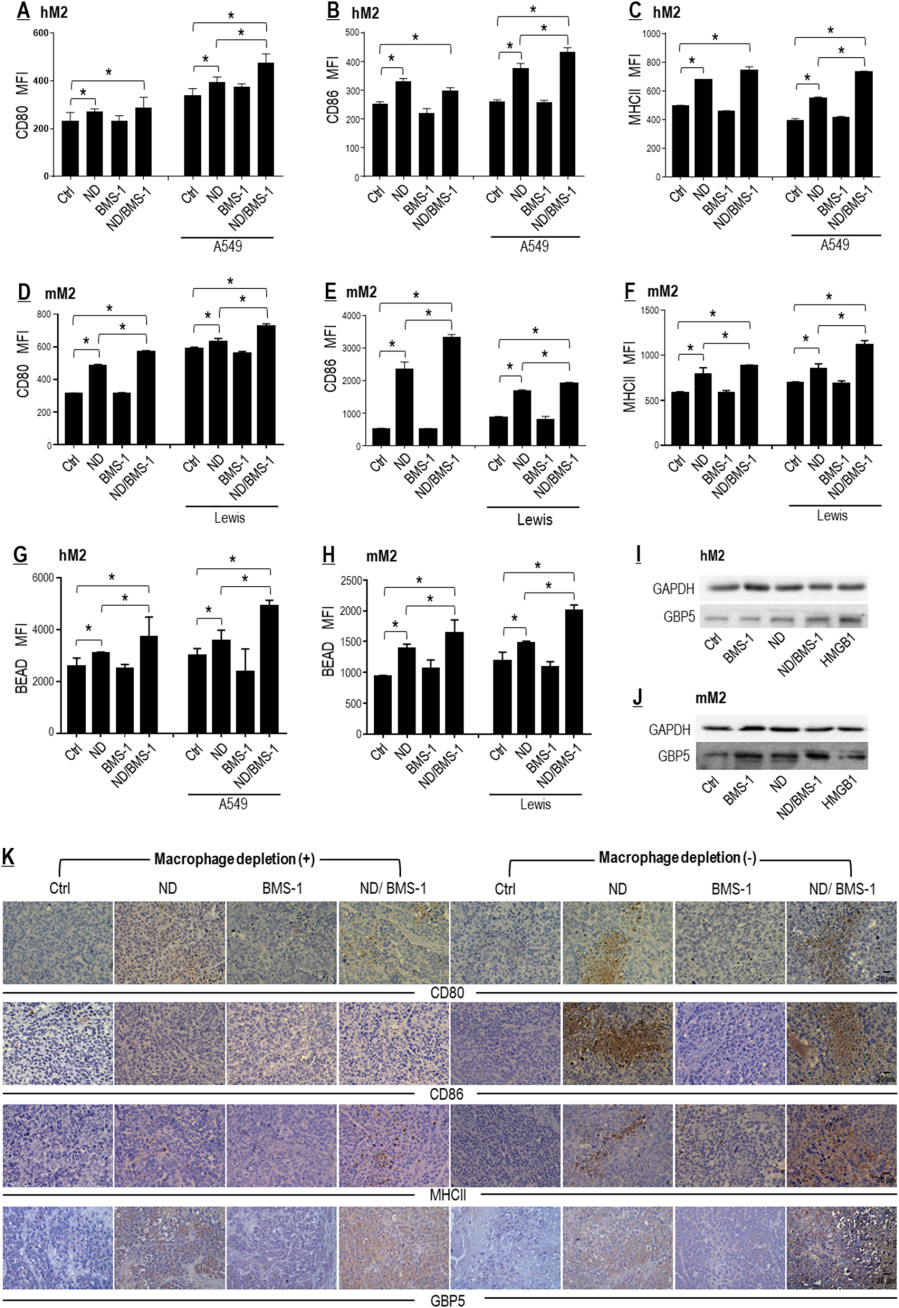
PD-L1 blockade by BMS-1 enhanced Nano-DOX-stimulated M1-like activation of TAMs. **A**–**C** BMS-1 promoted Nano-DOX**-**induced surface markers (CD80, CD86 & MHC-II) of M1-like activation in hM2 in the presence of A549 cells. **D**–**F** BMS-1 promoted Nano-DOX**-**induced surface makers (CD80, CD86 & MHC-II) of M1-like activation of mM2 both in the presence and absence of Lewis cells. **G**, **H** BMS-1 promoted Nano-DOX**-**induced phagocytosis of fluorescent latex beads by hM2 and mM2 both in the presence and absence of the cancer cells. **I**, **J** BMS-1 promoted Nano-DOX**-**induced GBP5 in single-cultured hM2 and mM2. Cell surface CD80, CD86 & MHC-II were assayed by FACS analysis of immunofluorescent staining and GBP5 protein was assayed by western blotting. **K** Nano-DOX treatment led to increased immunohistological staining of CD80, CD86, MHC-II and GBP5 in subcutaneous xenografts of Lewis cells in mice. FACS histogram geometric means were used to quantify mean fluorescence intensity (MFI). Values were means ± SD (*n* = 3, *p < *0.05*). Drug concentration was 2 μg/mL for DOX and Nano-DOX and 1 μM for BMS-1 in the in vitro experiments and treatment duration was 24 h. Representative FACS dot plots for A-H were provided in Additional file 1: Figure S6

Nano-DOX stimulated NSCLC cells to release DAMPs (Fig. 2) which are endogenous adjuvants capable of repolarizing the immunosuppressive and anti-inflammatory TAMs (M2-type) toward an immunostimulatory and pro-inflammatory phenotype (M1-type) [14]. However, there was concurrent induction of the immune checkpoint proteins PD-L1/PD-1 (Fig. 3), which was supposed to put a brake on the M1-like activation of the TAMs. We thus posited that blockade of the PD-L1/PD-1 interaction would take the brake off and thereby enhance Nano-DOX-stimulated M1-like activation of the TAMs. To test this postulate, we subjected human and murine TAM models (hM2 and mM2) in mixed culture with NSCLC cells to Nano-DOX alone or a combination of Nano-DOX and BMS-1. BMS-1 is a small molecule agent that induces PD-L1 dimerization and thereby blocks its interaction with PD-1 [23–25]. Indeed, both hM2 and mM2 displayed enhanced M1-like activation by the Nano-DOX/BMS-1 combination over Nano-DOX alone, as revealed by the analysis of M1 surface markers (CD80, CD86, and MHC-II), M2 surface marker (CD206), and phagocytic function (Fig. 6A–H; Additional file 1: Figure S6 I, J). The suggestion herein is that BMS-1 may promote Nano-DOX-induced M1-like activation of TAMs in a tumor cell-dependent manner, probably by blocking PD-L1/PD-1 interaction between the tumor cells and the TAMs.

Unexpectedly, Nano-DOX also induced M1-like activation in single-cultured hM2 and mM2 (Fig. 6A–H; Additional file 1: Figure S6 I, J). Additional evidence was the upregulated protein GBP5 (Fig. 6I, J) which is a sensitive indicator of macrophage M1 activation [36]. Intriguingly, BMS-1 also enhanced Nano-DOX-induced M1-like activation of single-cultured hM2 and mM2, and the enhancement was more conspicuous in mM2 (Fig. 6A–J; Additional file 1: Figure S6 I, J). The suggestion herein is that BMS-1 may enhance Nano-DOX-stimulated M1-like activation of TAMs in a tumor cell-independent manner. We postulated that this manner of action might be due to BMS-1 directly blocking PD-L1 in the TAMs as PD-L1 has recently been recognized as a negative regulatory signal of macrophage functions [37] and is upregulated upon M1 activation [38]. PD-L1 expression in the TAMs was thus explored and results are presented in “Nano-DOX induced PD-L1 in TAMs” section.

In vivo data overall agreed with the in vitro results, showing increased expression of CD80, CD86, MHC-II, and GBP5 indicative of M1-like activation in tumor grafts not depleted of macrophages (Fig. 6K).

#### Nano-DOX induced PD-L1 in TAMs

**Fig. 7:**
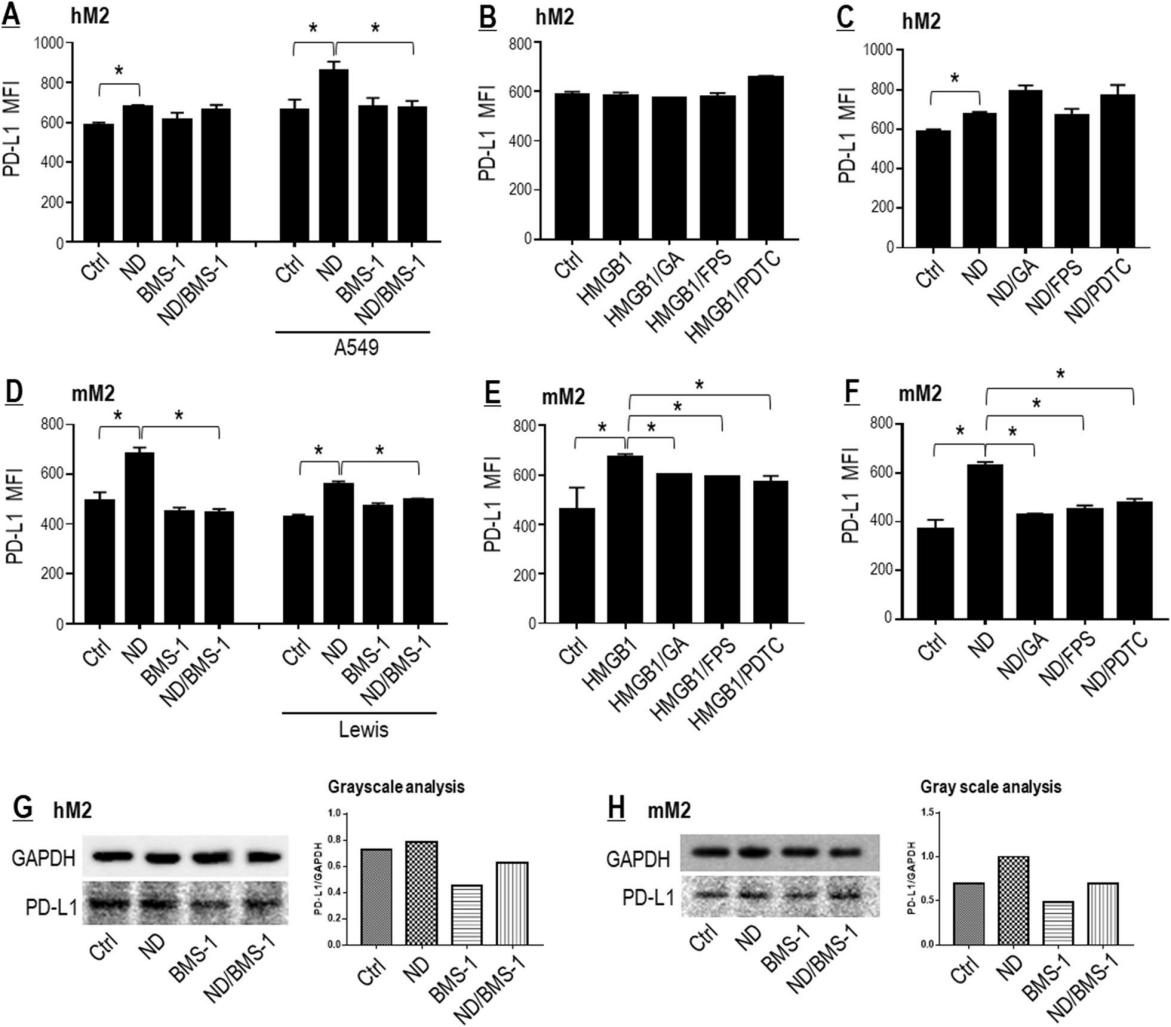
Nano-DOX induced PD-L1 in TAMs. **A** Nano-DOX induced cell surface PD-L1 in the hM2 both in the presence and absence of A549 cells. **B** Exogenously given HMGB1 did not induce cell surface PD-L1 in the hM2 and blockers of the HMGB1/RAGE/NF-κB pathway had little effect on cell surface PD-L1 expression. **C** Blockers of the HMGB1/RAGE/NF-κB pathway did not suppress Nano-DOX-induced cell surface PD-L1 in the hM2. **D** Nano-DOX induced cell surface PD-L1 in the mM2 both in the presence and absence of Lewis cells. **E** Exogenously given HMGB1 induced cell surface PD-L1 in the mM2 and this effect could be alleviated by blockers of the HMGB1/RAGE/NF-κB pathway. **F** Blockers of the HMGB1/RAGE/NF-κB pathway suppressed Nano-DOX-induced cell surface PD-L1 in the hM2. **G**, **H** Nano-DOX increased PD-L1 protein both in the hM2 and mM2. Cell surface PD-L1 was assayed by FACS analysis of immunofluorescent staining. PD-L1 protein was assayed by western blotting and grayscale analysis of the blot strips was performed. FACS histogram geometric means were used to quantify mean fluorescence intensity (MFI). Values were means ± SD (*n* = 3, *p < *0.05*). Drug concentration was 2 μg/mL for DOX and Nano-DOX and 1 μM for BMS-1 in the in vitro experiments and treatment duration was 24 h. Representative FACS dot plots for **A**–**F** were provided in Additional file 1: Figure S7

As shown in Fig. 7A, D, Nano-DOX indeed induced PD-L1 in hM2 and mM2 both in the presence and absence of the cancer cells. But PD-L1 induction by Nano-DOX in the hM2 was independent of the HMGB1/RAGE/NF-κB pathway as HMGB1 failed to induce PD-L1 (Fig. 7B) and blockers of the pathway did not repress Nano-DOX-induced PD-L1 (Fig. 7C). In the case of mM2, Nano-DOX-induced PD-L1 appeared to involve the HMGB1/RAGE/NF-κB pathway as HMGB1 markedly induced PD-L1 (Fig. 7E) and blockers of the pathway repressed both HMGB1- and Nano-DOX-induced PD-L1 (Fig. 7F). Of note, PD-L1 induction by Nano-DOX was more acute in the mM2 than in the hM2 in single culture (Fig. 7A, C, D, F–H), which may at least partly explain why the synergetic M1-like activation by Nano-DOX and BMS-1 was more conspicuous in the mM2 (Fig. 6).

### PD-L1 blockade and Nano-DOX achieved synergistic anti-NSCLC efficacy

#### BMS-1 enhanced Nano-DOX’s suppression of tumor growth both in a TAM-dependent and TAM-independent manner

**Fig. 8:**
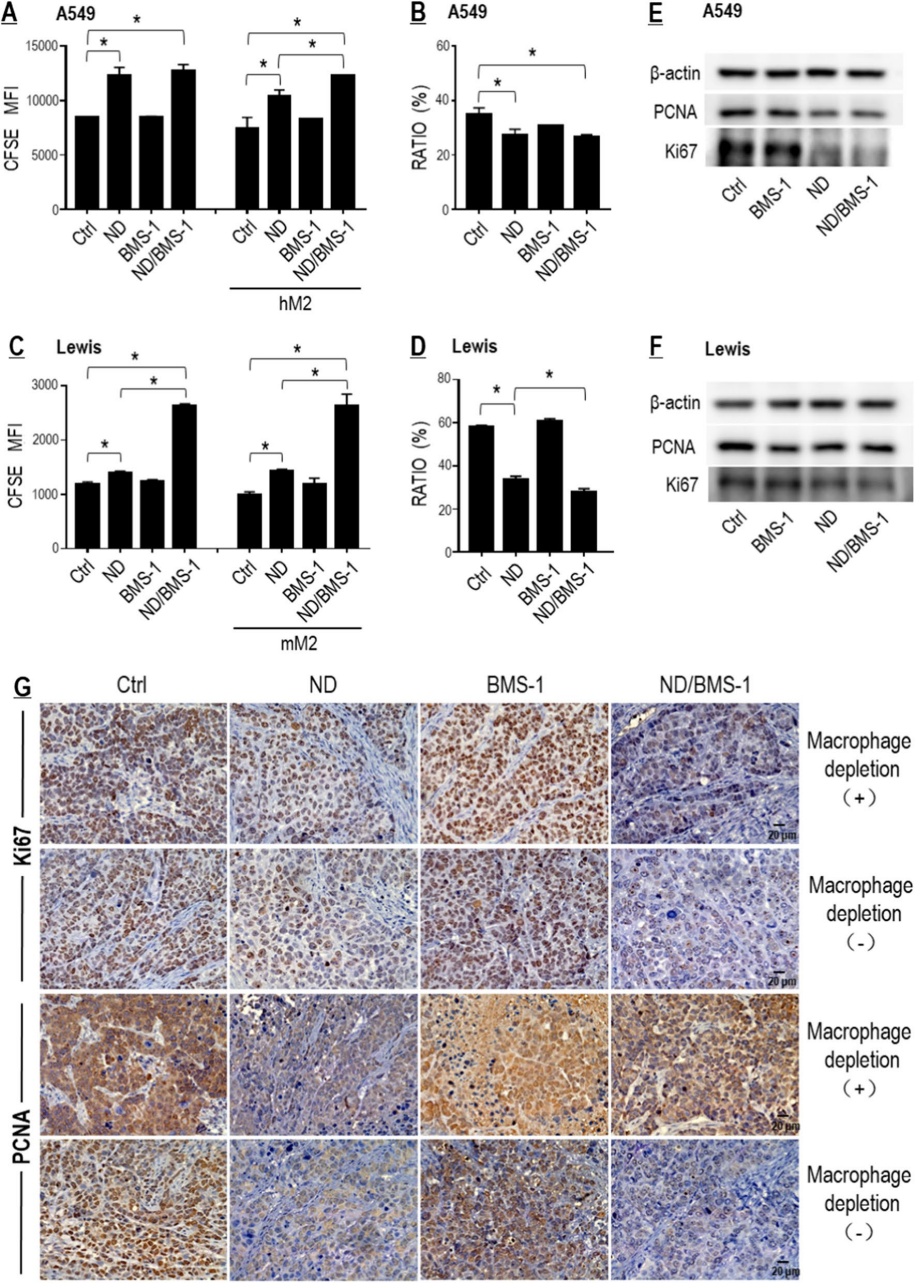
BMS-1 and Nano-DOX synergistically inhibited proliferation of lung cancer cells. **A** BMS-1 enhanced Nano-DOX’s action to inhibit A549 cell growth only in mixed culture with the hM2. **B** Proportion of A549 cells in the mixed culture. **C** BMS-1 enhanced Nano-DOX’s action to inhibit Lewis cell growth both in single culture and in co-culture with the mM2. **D** Proportion of Lewis cells in the mixed culture. **E**, **F** Effects of Nano-DOX, BMS-1 and the combination thereof on the protein levels Ki67 and PCNA in the in vitro A549 and Lewis cells. **G** Nano-DOX treatment led to decreased immunohistological staining of Ki67 and PCNA in subcutaneous xenografts of Lewis cells in mice. Cell growth was assayed by FACS analysis of decay of CFSE staining. Ki67 and PCNA protein was assayed by western blotting. FACS histogram geometric means were used to quantify mean fluorescence intensity (MFI). Values were means ± SD (*n* = 3, *p < *0.05*). Drug concentration was 2 μg/mL for DOX and Nano-DOX and 1 μM for BMS-1 in the in vitro experiments and treatment duration was 24 h. Representative FACS zebra plots for **A**–**D** were provided in Additional file 1: Figure S8

As BMS-1 and Nano-DOX were demonstrated to achieve synergistic M1-like activation of TAMs (Fig. 6), synergistic anti-NSCLC action was then expected of combined use of the two drugs in a TAM-dependent manner. For proof thereof, we first checked the proliferation of tumor cells (A549 & Lewis) in mixed-culture with the TAM models (hM2 & mM2). Decay of CFSE staining was assayed to indicate tumor cell proliferation. The proportion of tumor cells in the mixed culture was also calculated to reflect proliferation. As expected, Nano-DOX suppressed proliferation both of A549 and Lewis cells in mixed culture with their corresponding TAM models and BMS-1 enhanced Nano-DOX’s effect in the mixed culture setting (Fig. 8A–D). Surprisingly, BMS-1 also markedly enhanced Nano-DOX’s suppression of Lewis cell proliferation in the single culture (Fig. 8C). Down-regulation of Ki67, a marker of cell proliferation, also suggests BMS-1/Nano-DOX synergy in the single cultured tumor cells (Fig. 8E, F), which was also reflected in the in vivo study (Fig. 8G). These observations indicate that BMS-1 may act in synergy with Nano-DOX to inhibit tumor cell growth both in a TAM-dependent and TAM-independent manner. The TAM-independent synergy, we posit, probably stems from BMS-1 directly blocking the PD-L1 induced by Nano-DOX in the tumor cells.

#### BMS-1 enhanced Nano-DOX’s tumoricidal activity mainly in a TAM-dependent manner

**Fig. 9:**
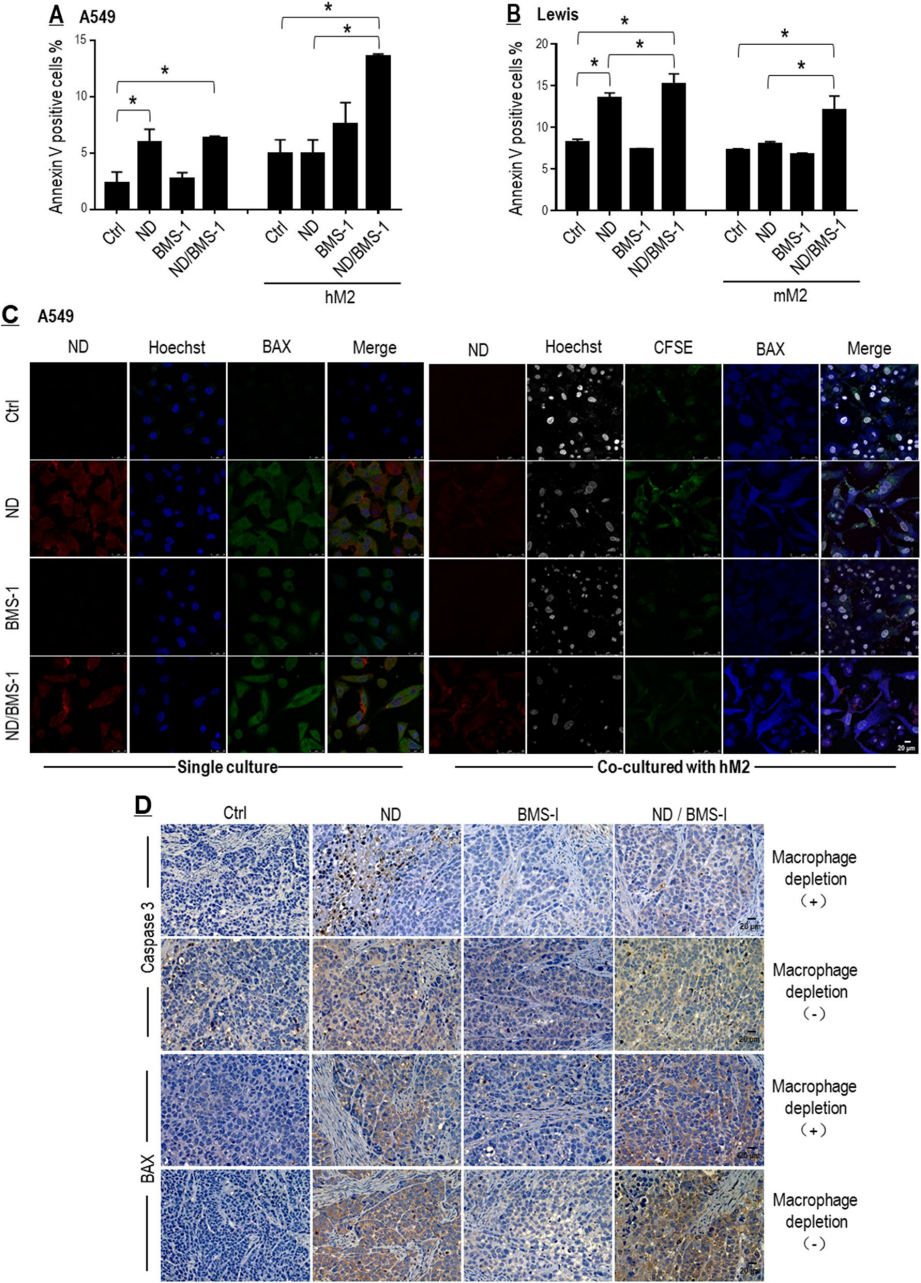
BMS-1 and Nano-DOX synergistically promoted apoptosis of lung cancer cells. **A**, **B** BMS-1 potentiated Nano-DOX’s action to induce apoptosis of cancer cells (A549 & Lewis) mainly in mixed culture with the TAM models (hM2 & mM2). Apoptosis was indicated by the cell surface presence of annexin v. **C** BMS-1 potentiated Nano-DOX’s action to induce BAX in the A549 cells mainly in mixed culture with the hM2. **D** Nano-DOX treatment led to increased immunohistological staining of caspase 3 and BAX in subcutaneous xenografts of Lewis cells in mice. Cell surface annexin v was assayed by FACS analysis of immunofluorescent staining. BAX expression was assayed by confocal microscopy of immunofluorescent staining. FACS histogram geometric means were used to quantify mean fluorescence intensity (MFI). Values were means ± SD (*n* = 3, *p < *0.05*). Drug concentration was 2 μg/mL for DOX and Nano-DOX and 1 μM for BMS-1 in the in vitro experiments and treatment duration was 24 h. Representative FACS zebra plots for **A** and **B** were provided in Additional file 1: Figure S9

Tumor cell apoptosis was next evaluated by checking cell surface annexin-v and BAX expression. As shown in Fig. 9A–C, Nano-DOX markedly increased apoptosis of both A549 and Lewis cells either in a single culture or in mixed culture with the cancer cells, but BMS-1 enhanced Nano-DOX’s effect mainly in the mixed culture setting. In vivo experiment also showed a greater extent of apoptosis in tumor grafts not depleted of macrophages (Fig. 9D). These observations suggest that BMS-1 may enhance Nano-DOX-induced tumor cell killing primarily in a TAM-dependent manner.

#### BMS-1 potentiated Nano-DOX’s therapeutic efficacy against graft tumors in a TAM-dependent manner

**Fig. 10:**
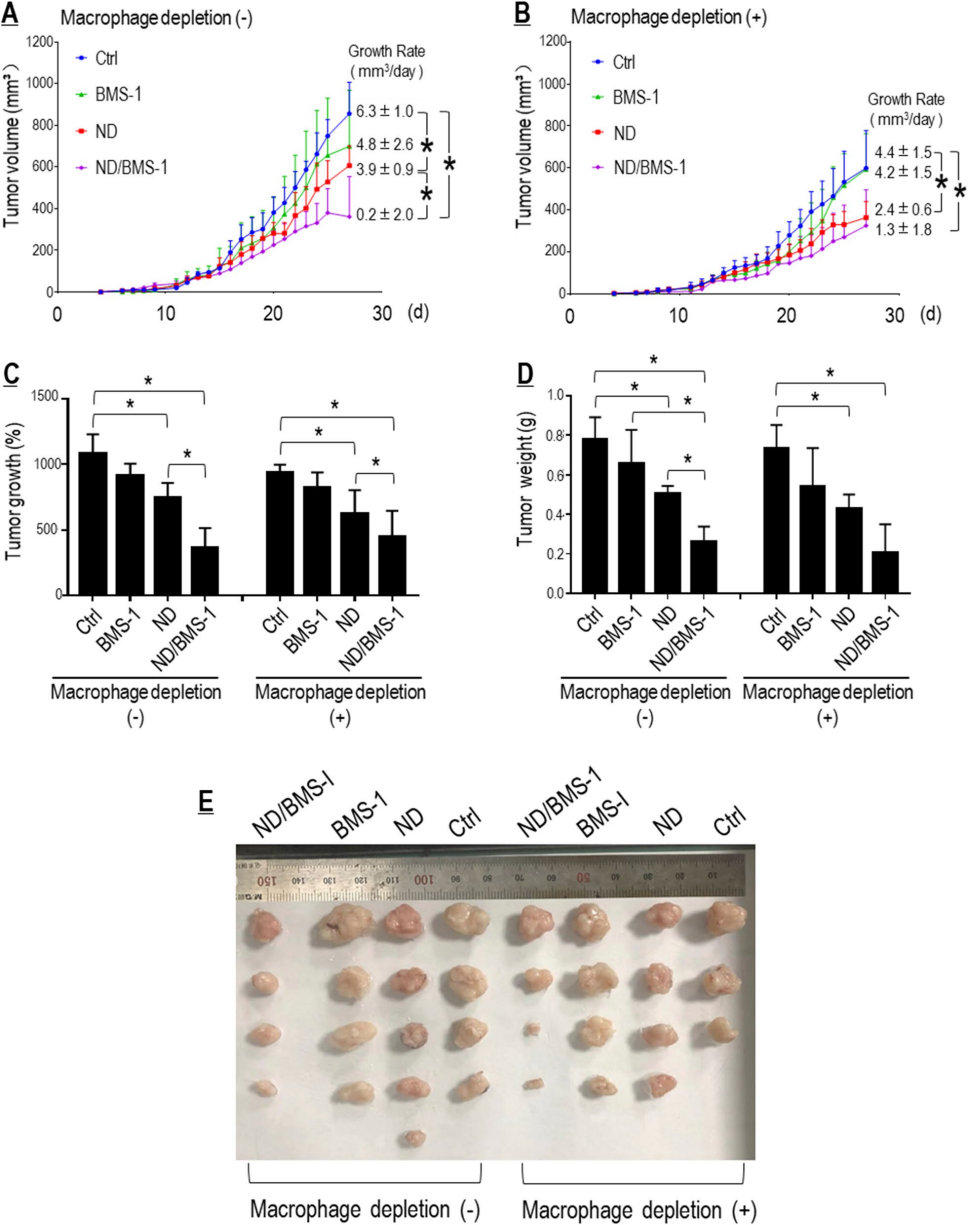
PD-L1 blockade potentiated Nano-DOX’s therapeutic efficacy against subcutaneous grafts of Lewis cells. **A**, **B** Growth curves of Lewis tumor xenografts with or without TAM depletion. Growth rates over the treatment duration (from day 13 to day 27) were calculated by regression analysis. **C** Tumor growth over the treatment duration, which is expressed as tumor volume on the last day as the percentage of tumor volume on the first day in a mouse. **D** Tumor weight at the time of sacrifice. **E** Dissected tumor grafts after sacrifice. Values were means ± SD (*n* = 3, *p < *0.05*)

Finally, the therapeutic synergy of Nano-DOX and BMS-1 was demonstrated in vivo on subcutaneous Lewis tumor grafts with or without TAM depletion. The distribution of Nano-DOX in the tumor grafts was confirmed by ex vivo fluorescent imaging and fluorescent microscopy of tumor tissue sections (Additional file 1: Figures S11, S12). As shown in Fig. 10A–E, tumor grafts depleted of TAMs exhibited slower growth and lower tumor weight at the time of sacrifice than those without TAM depletion, indicating the pro-tumor role of TAMs. Nano-DOX significantly slowed tumor growth irrespective of TAM depletion. The impact of BMS-1 solo on tumor growth was marginal, but slightly more appreciable in tumor grafts without TAM depletion. BMS-1 markedly potentiated Nano-DOX’s suppressive efficacy primarily in tumor grafts without TAM depletion. These observations strongly suggest that PD-L1 blockade could enhance Nano-DOX’s anti-NSCLC therapeutic efficacy in a TAM-dependent manner.

## Discussion

In the present work, we had set out to investigate two major hypotheses: ➀ Nano-DOX could stimulate tumor cells to release HMGB1 which will act through the RAGE receptor to promote NF-κB-dependent PD-L1 expression in the tumor cells and PD-1 expression in the TAMs; and ➁ blockade of Nano-DOX-induced PDL-1 in the tumor cells by BMS-1 will enhance TAM-mediated anti-tumor immune response stimulated by Nano-DOX, thus achieving therapeutic synergy with Nano-DOX. As it turned out, we obtained findings that not only validate these hypotheses but also expand their notions.

On the side of the tumor cells, Nano-DOX is found to induce RAGE expression at the same time of stimulating HMGB1 secretion in the NSCLC cells, thus reinforcing PD-L1 upregulation by the autocrine HMGB1 as well as paracrine HMGB1 derived from the TAM. This self-reinforcing mechanism of autocrine and paracrine PD-L1 upregulation is a discovery that enriches the notion of our first starting hypothesis. The mechanisms whereby Nano-DOX induces HMGB1 secretion and RAGE expression are under further investigation. Remarkably, RAGE induction by Nano-DOX appears not to be dependent on HMGB1. The PD-L1 upregulation in the tumor cells has double-count significance. First, the upregulated PD-L1 in the tumor cells poses an extrinsic check over the tumor-infiltrating immune cells e.g. the TAMs and T-lymphocytes via engagement with PD-1 as the role of PD-L1/PD-1 ligation in suppressing immune responses of antigen-presenting cells and T-cells has been well established [1, 3]. Blocking the PD-L1/PD-1 ligation would relieve the check thus unleashing the anti-tumor activities of these immune cells. As proof thereof, we have shown that the PD-L1 blocker BMS-1 can enhance Nano-DOX-stimulated M1-type repolarization of TAMs by negating the inhibition of co-cultured NSCLC cells. This finding also substantiates our second starting hypothesis. Second, but not of secondary importance, the upregulated PD-L1 also represents an increased pro-survival signal intrinsic to the tumor cells. There is emerging evidence that cell surface PD-L1 is upregulated under cell stress and transduces survival signals in tumor cells to promote cancer growth, metastasis, and resistance to therapy [39, 40]. Hence, blocking the induced PD-L1 in the tumor cells would undermine tumor survival and growth and this idea finds substantiation in our observation that PD-L1 blockade by BMS-1 markedly enhanced Nano-DOX’s suppression of Lewis cell proliferation independent of the repolarized mM2. This synergy between PD-L1 blockade and Nano-DOX is intrinsic to the tumor cells and represents a new dimension to the notion of our second starting hypothesis.

On the side of the TAMs, Nano-DOX was found to induce PD-1 also via activation of the HMGB1/RAGE/NF-κB axis. The HMGB1 can be autocrine i.e. from the TAMs, or paracrine i.e. from the tumor cells. Unlike in the tumor cells, there is no concurrent induction of RAGE. Both human and mouse TAMs have recently been found to express PD-1 which has effector functions both extrinsic and intrinsic to the TAMs. Intrinsically, PD-1 expression per se probably represents a phagocytically repressed state of the TAMs [8, 41]. Extrinsically, PD-1 serves as a handle that could be used by the tumor cells to curb TAMs’ immune function, particularly the phagocytic potency, via ligation of PD-L1 [42]. Thus, PD-1 provides a promising target to stimulate the anti-tumor activity of TAMs. Gordon et al. showed that blockade of tumor cell PD-L1 ligation to TAM PD-1 could restore TAM phagocytosis and promote anti-tumor efficacy by the TAMs [8]. In line with these observations, we have demonstrated that blockade of Nano-DOX-induced PD-L1 in the NSCLC cells enhanced the phagocytic potency and anti-tumor activity of co-cultured TAMs. Intriguingly, Nano-DOX was also found to induce PD-L1 in the TAMs. In line with this observation, BMS-1 enhanced Nano-DOX-induced M1-type activation of the TAMs, independent of the tumor cells. This is compelling evidence that BMS-1 abolishes the intrinsic inhibitory signal of PD-L1 induced by Nano-DOX in the TAMs. These findings represent another significant advance on the notions of our starting hypotheses and hold therapeutic significance in that they demonstrate that PD-L1 is an inducible anti-M1 polarization signal intrinsic to the TAMs, which can be targeted for therapeutic modulation of TAM phenotype. In agreement with our findings, Hartley et al. recently demonstrated PD-L1 to be a constitutive negative signal that drives macrophages towards an immune-suppressive cell phenotype which could be reversed by PD-L1 antibodies thus triggering macrophage-mediated antitumor activity [37]. PD-L1 induction by Nano-DOX appears to be dependent on HMGB1 in the mM2 but not hM2 and causes of the difference await elucidation. It should be noted that the mM2 are derived from bone marrow precursor cells whereas the hM2 are derived from the THP leukemia cells, which may behave differently than natural type-2 macrophages. Species difference might also play an underlying role herein.

It is a highlighted discovery that BMS-1 potentiated Nano-DOX’s therapeutic efficacy against NSCLC in a manner that depends on the synergistic repolarization of the pro-tumor type-2 TAMs into the anti-tumor M1 phenotype by Nano-DOX and BMS-1. In vitro experiments demonstrated that the synergistic repolarization of TAMs probably works at two dimensions. On one hand, Nano-DOX induces both PD-L1 in the tumor cells and PD-1 in the TAMs. Ligation of tumor cell PD-L1 with TAM PD-1 works as an external brake on TAM repolarization. BMS-1 blocks PD-L1/D-1 ligation thus removing the external brake on TAM repolarization. On the other hand, Nano-DOX could repolarize the TAMs by itself or via stimulating the tumor cells’ immunogenicity. But there is a concurrent induction of PD-L1 in the TAMs serving as an intrinsic brake on TAM repolarization. BMS-1 also blocks the induced PD-L1 thus removing the intrinsic brake, leading to enhanced TAM repolarization. The repolarized TAMs both kill the tumor cells and suppress their growth. It must be noted that BMS-1 could also potentiate Nano-DOX’s action to suppress tumor cell growth via blocking Nano-DOX-induced PD-L1 in the tumor cells. This effect is independent of the TAMs and demonstrated in the in vitro experiments, albeit not reflected in the in vivo therapeutic efficacy.

Nano-DOX was originally designed as a delivery form of DOX for targeted tumor chemotherapy [22]. Work later on showed Nano-DOX to have properties fundamentally different than DOX, with the most outstanding being a much reduced cytocidal potency than seen with DOX [43]. DOX is a typical tumoricidal agent with severe toxicity to bone marrow and the immune system. However, Nano-DOX has been found largely to arrest cell proliferation rather than to induce cell death [16, 22, 43]. In other words, Nano-DOX’s cytotoxicity manifests as growth inhibition rather than cell killing. Cancer cells are proliferative but the TAMs are not, which explains the discrepant toxicity results of cancer cells and TAMs shown in Additional file 1: Figure S1. We have previously used immune cells e.g. monocytes, macrophages, and dendritic cells, which are typically very sensitive to DOX’s toxicity, as active carriers for tumor-targeted delivery of Nano-DOX which subsequently reprogrammed the tumor immune microenvironment towards an anti-tumor phenotype [14, 15, 44]. In the present work, Nano-DOX was demonstrated to induce PD-L1 in the NSCLC cells via activation of the HMGB1/RAGE/NF-κB axis. DOX was also found to induce PD-L1 in the same NSCLC models, but obviously via mechanisms other than the HMGB1/RAGE/NF-κB pathway. These discrepancies may arise from disparate cell damage profiles induced by the two forms of doxorubicin. In a separate work on murine breast cancer cells [45], we demonstrate that DOX is distributed both in the nuclei and lysosomes causing both severe DNA damage and endoplasmic reticulum (ER) stress while Nano-DOX mainly stays in the lysosomes where doxorubicin is slowly released, due to acid hydrolysis of the hydrazone bond, to the cytoplasm inducing endoplasmic reticulum stress but insubstantial DNA damage. These observations may also underlie Nano-DOX’s lower cytotoxicity than DOX. Further investigations on the NSCLC models are underway to elucidate the mechanisms of Nano-DOX’s PD-L1/PD-1-inducing action upstream to the autocrine secretion of HMGB1. A look was also taken at the vital organ distribution of Nano-DOX and systemic toxicity of Nano-DOX, BMS-1, and Nano-DOX plus BMS-1. The liver appeared to be a major accumulation site of Nano-DOX besides the tumors (Additional file 1: Figure S11). Interestingly, macrophage depletion seemed to reduce liver accumulation of Nano-DOX (Additional file 1: Figure S11), which is understandable as macrophages in the reticuloendothelial system, particularly the liver, play a key role in the clearance of particles in the blood circulation. Macroscopic and IHC examinations did not reveal any significant tissue damage of the vital organs in all treated animals (Additional file 1: Figures S13, S14). All treated animals not depleted of macrophages gained weight at similar rates to control during the treatment duration while animals depleted of macrophages gained weight at lower rates to their counterparts without macrophage depletion, probably due to toxicity of the macrophage depletion agent (Additional file 1: Figure S15).

**Fig. 11:**
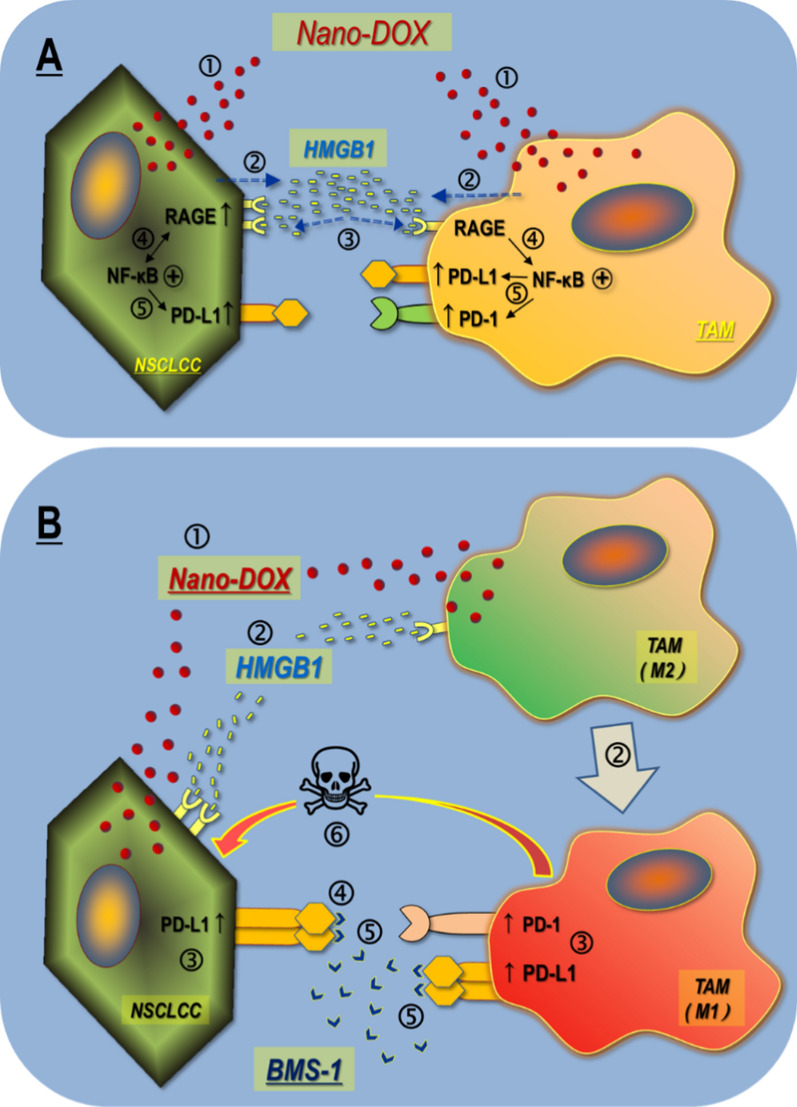
Main findings of this work. **A** Nano-DOX induce PD-L1 and PD-1 in the tumor cell-macrophage interaction through autocrine and paracrine activation of the HMGB1/RAGE/NF-κB pathway. ➀ & ➁ Nano-DOX stimulates HMGB1 release from the NSCLCC & TAM. ➂ & ➃ HMGB1 binds with RGAE to activate NF-κB in the NSCLCC & TAMs. NF-κB activation also upregulates RAGE in the NSCLCC. ➄ Activated NF-κB upregulates PD-L1 in the NSCLCC. Activated NF-κB upregulates PD-L1 & PD-1 in the TAMs. **B** PD-L1 blocker BMS-1 enhances Nano-DOX-stimulated M1-like activation (anti-tumor phenotype) of TAMs via blocking PD-L1 induced by Nano-DOX in the lung cancer cells (NSCLC) and TAMs. ➀ Nano-DOX stimulates HMGB1 release from the NSCLCC & TAMs. ➁ HMGB1 bind with RAGE in the NSCLCC and TAMs. TAMs are repolarized to M1-like phenotype. ➂ PD-L1 are upregulated in the NSCLCC. Both PD-L1 and PD-1 are upregulated in the TAMs. ➃ BMS-1 blocks PD-L1 in the NSCLCC leading to growth suppression. ➄ BMS-1 prevents NSCLCC PD-L1 from binding with TAM PD-1 and blocks PD-L1 in the TAMs, thus enhancing M1-like TAM activation. ➅ M1-like TAMs suppress NSCLCC growth and induce apoptosis

The primary novelty of this work is the discovery that the autocrine and paracrine HMGB1/RAGE/NF-κB signaling is a key mechanism for upregulation of PD-L1 and PD-1 in the tumor cell-TAM interaction, which can be activated by Nano-DOX. Based on this discovery, we have further demonstrated that (1) blockade of the induced PD-L1 in the NSCLC cells not only abolishes their suppression of the TAMs, but also disrupts PD-L1’s pro-survival function intrinsic to the tumor cells, and (2) blockade of the induced PD-L1 in the TAMs cancels PD-L1’s intrinsic suppressive function resulting in enhanced anti-tumor M1-like activation. These findings, as summarized in Fig. 11, represent the basis of a new immunotherapy strategy of cancer based on PD-L1/PD-1 blockade, which stimulates powerful antitumor immune response mediated by TAMs instead of lymphocytes. This strategy will be particularly beneficial to patients with “cold tumors” which are characterized by poor lymphocyte infiltration and lymphocyte exhaustion. It is also worth noting that although there have been increasing reports of a therapeutic synergy between chemotherapy [46–49], be it in nano-form or not, and checkpoint blockade therapy, elucidation of the underlying mechanisms is lacking. The present work is among the few studies that provide a compelling mechanistic rationale for the combinatorial use of chemotherapy and checkpoint blockade.

## Conclusions

PD-L1/PD-1 upregulation mediated by autocrine and paracrine activation of the HMGB1/RAGE/NF-κB signaling is a key response of lung cancer cells and their TAMs to stress, which can be induced by Nano-DOX. Blockade of Nano-DOX-induced PD-L1, both in the cancer cells and the TAMs, achieves enhanced activation of TAM-mediated anti-tumor response.

## Supplementary Information

**Additional file 1: Figure S1.** Effects of polyglycerol-coated nanodiamonds (PG-Nd), Nano-DOX (ND), BMS-1 and doxorubicin (DOX) on viability of lung cancer cells and TAM models. **Figure S2, S4, S5, S6, S7, S8, S9.** Representative FACS dot plots for data. **Figure S3.** Nano-DOX and DOX induced PD-L1 in the NSCLC cells. **Figure S10.** Macrophage depletion in the tumors confirmed by IHC analysis of macrophage surface marker CD11b. **Figure S11.** Ex vivo imaging showing drug fluorescence in tumor xenografts and vital organs. **Figure S12.** Fluorescent imaging of tumor shows the presence of Nano-DOX. Blue fluorescence is nuclear staining of DAPI. Red fluorescence comes from Nano-DOX (ND). **Figure S13.** Tumor xenografts and vital organs excised from sacrificed animals. **Figure S14.** H&E staining of major organs from the mice. **Figure S15.** Body weight curves of tumor bearing mice.

## Data Availability

All data generated or analyzed during this research are included in this manuscript.

## Abbreviations

ATP: Adenosine triphosphate
CRT: Calreticulin
DOX: Doxorubicin
EP: Ethyl pyruvate
FACS: Flow cytometry
GA: Glycyrrhizic acid
hM2: Human TAM model with type-2 phenotype
HMGB1: High mobility group box 1
HSP90: Heat shock protein 90
IHC: Immunohistochemical
LIPOSOMA: Liposome chlorophosphite
mM2: Mouse TAM model with type-2 phenotype
Nano-DOX: Nanodiamond-polyglycerol-doxorubicin conjugates
NSCLC: Non-small cell lung cancer
NF-κB: Nuclear factor-κB
PD-L1: Programmed death-ligand 1
PD-1: Programmed cell death-1
PDTC: Pyrrolidine dithiocarbamate
rHMGB1: Recombinant HMGB1
RAGE: Receptor for advanced glycation endproducts
TME: Tumor microenvironment
TAMs: Tumor-associated macrophages
UVR: Ultraviolet radiation
