# Supplementary material for: Synergy of nanodiamond–doxorubicin conjugates and PD-L1 blockade effectively turns tumor-associated macrophages against tumor cells
Source: J Nanobiotechnology. 2021 Sep 6;19:268. doi: 10.1186/s12951-021-01017-w (PMC8422639; doi:10.1186/s12951-021-01017-w)
Supplement: Supplementary file 1 — Additional file 1: Figure S1. Effects of polyglycerol-coated nanodiamonds (PG-Nd), Nano-DOX (ND), BMS-1 and doxorubicin (DOX) on viability of lung cancer cells and TAM models. Figure S2, S4, S5, S6, S7, S8, S9. Representative FACS dot plots for data. Figure S3. Nano-DOX and DOX induced PD-L1 in the NSCLC cells. Figure S10. Macrophage depletion in the tumors confirmed by IHC analysis of macrophage surface marker CD11b. Figure S11. Ex vivo imaging showing drug fluorescence in tumor xenografts and vital organs. Figure S12. Fluorescent imaging of tumor shows the presence of Nano-DOX. Blue fluorescence is nuclear staining of DAPI. Red fluorescence comes from Nano-DOX (ND). Figure S13. Tumor xenografts and vital organs excised from sacrificed animals. Figure S14. H&E staining of major organs from the mice. Figure S15. Body weight curves of tumor bearing mice. [file 12951_2021_1017_MOESM1_ESM.docx]

**Additional materials**

**
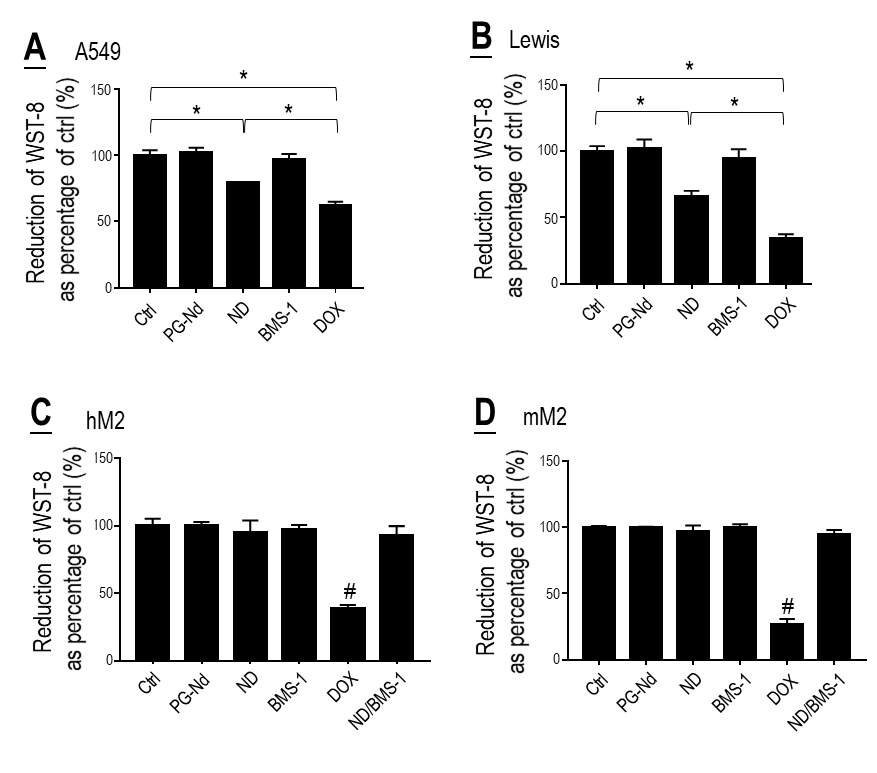
**

**Figure S1. Effects of polyglycerol-coated nanodiamonds (PG-Nd), Nano-DOX (ND), BMS-1 and doxorubicin (DOX) on viability of lung cancer cells (A, B) and TAM models (C, D).** Drug concentration was 2 μg/mL for DOX and Nano-DOX, 24 μg/mL for PG-Nd and 1μM for BMS-1. Treatment duration was 24 hr. Values are means ± SD (*n = 3, *p < 0.05*; *#p < 0.05*, compared with all other groups.)


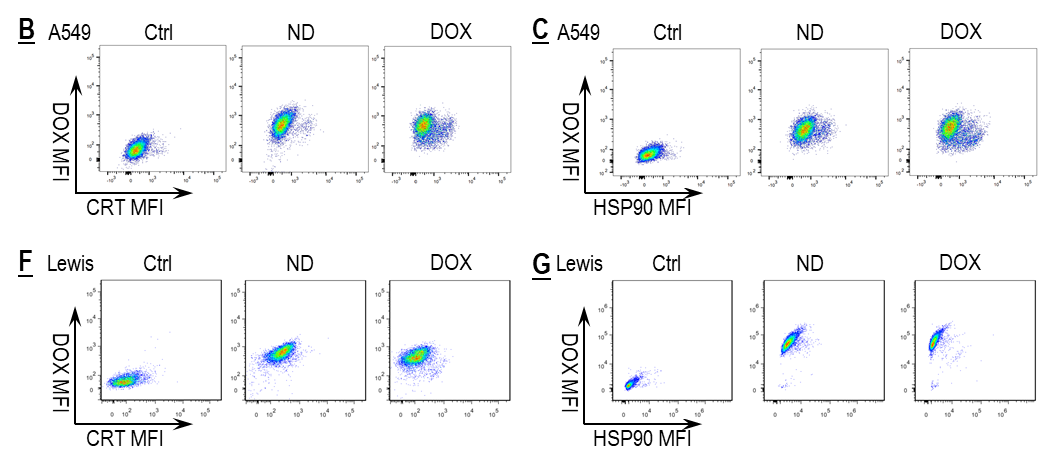


**Figure S2.** **Representative FACS dot plots for data presented in Fig. 2 B, C, F and G.**

**
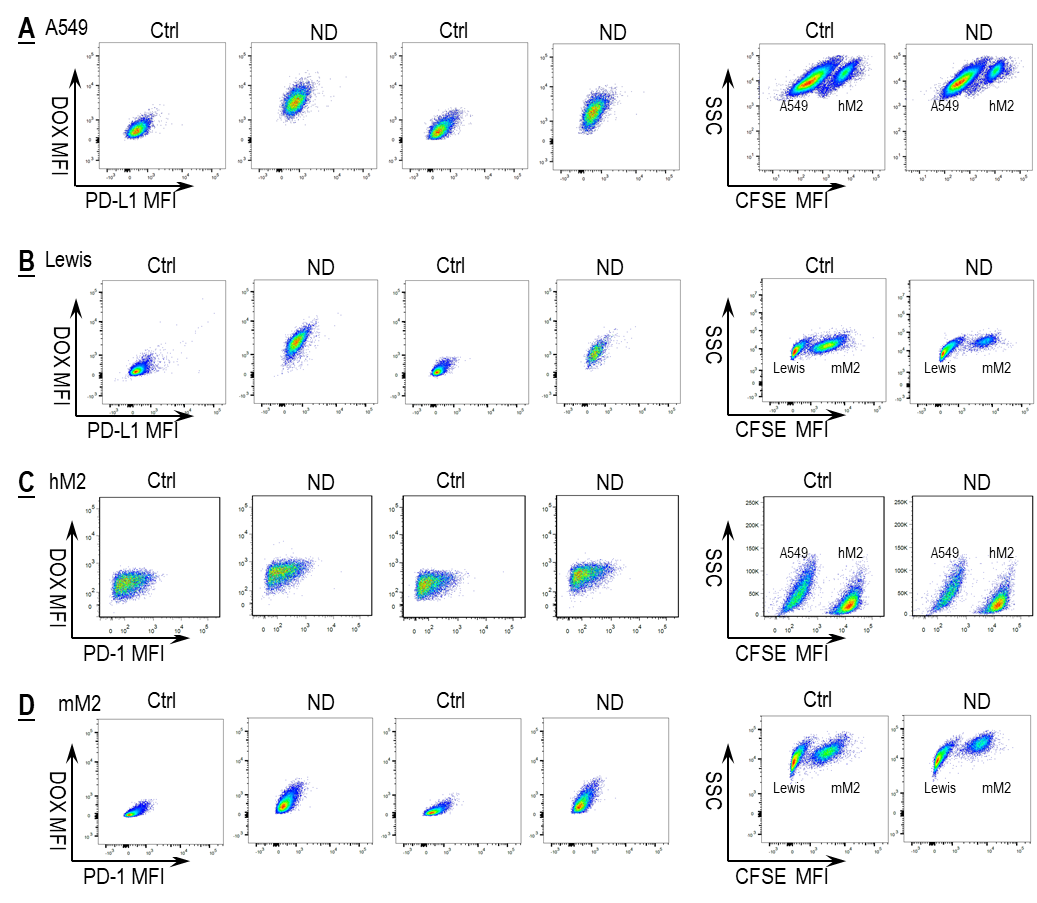
**

**Figure S3. A-D: Representative FACS dot plots for data presented in Fig. 3 A-D.**

**
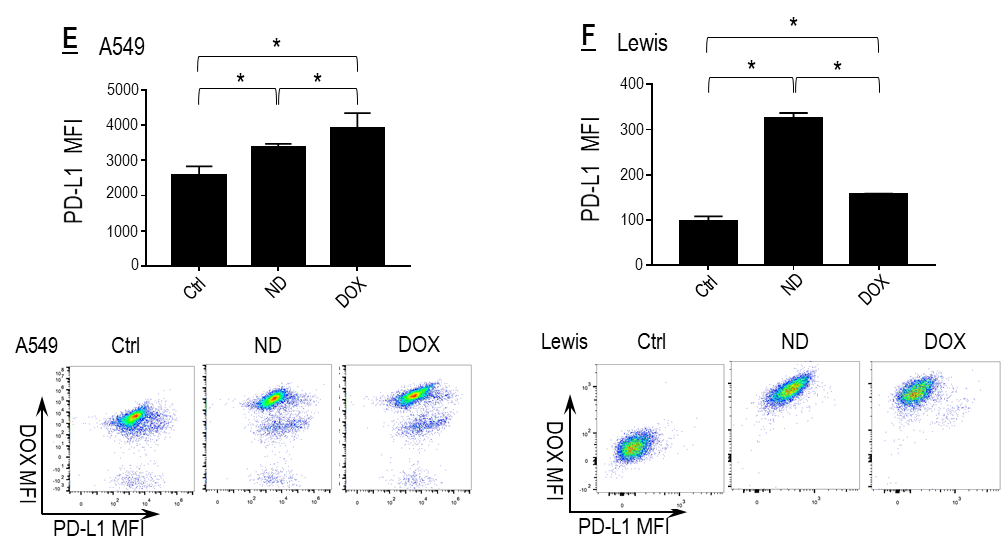
**

**Figure S3. E, F: Nano-DOX and DOX induced PD-L1 in the NSCLC cells.** Cell surface PD-L1 was assayed by FACS analysis of immunofluorescent staining. FACS histogram geometric means were used to quantify mean fluorescence intensity (MFI). Geometric means were used to quantify mean fluorescence intensity (MFI). Values were means ± SD (*n = 3, *p < 0.05*). Drug concentration was 2 μg/mL for DOX and Nano-DOX. Treatment duration was 24 hr.

**
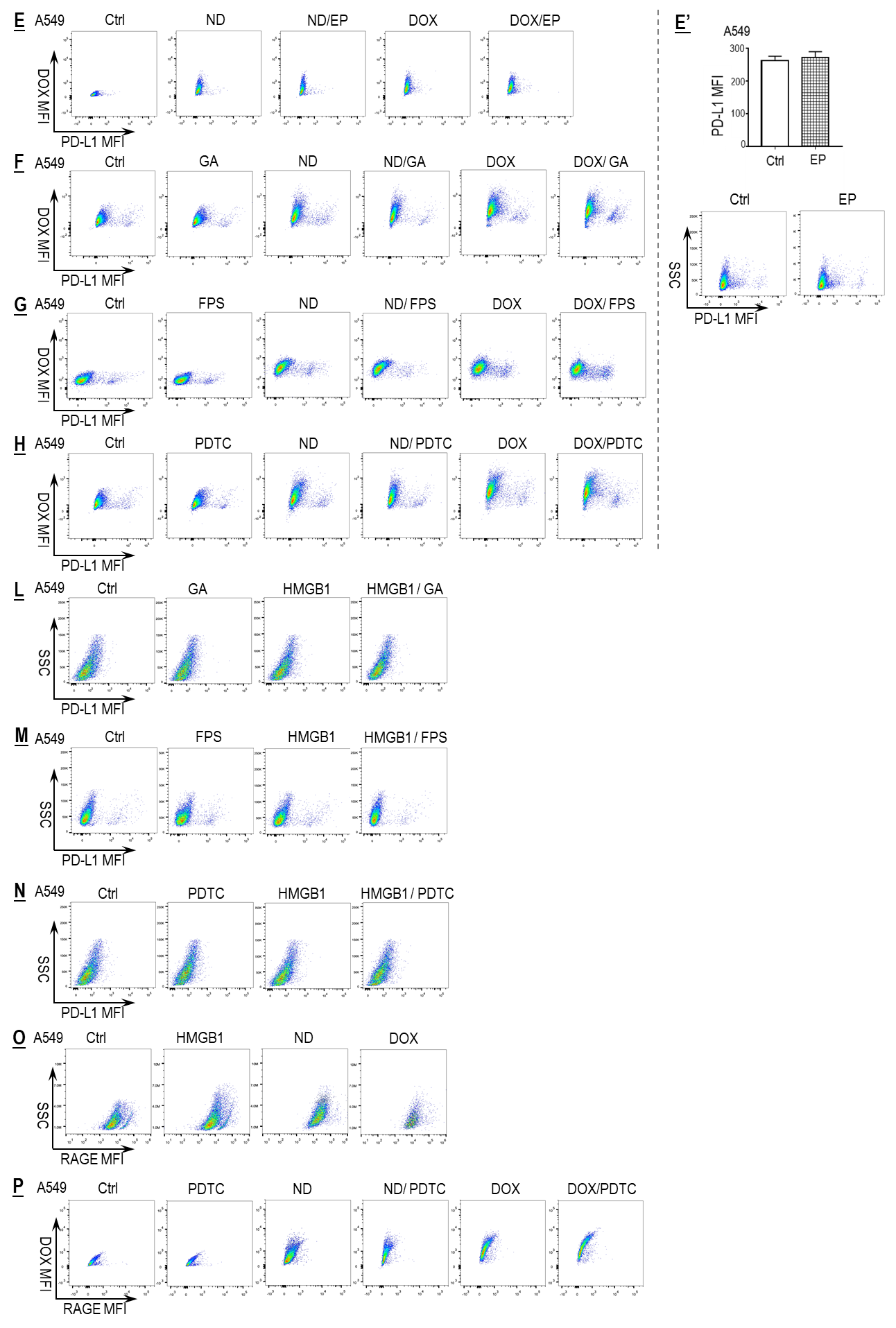
**

**Figure S4. Representative FACS dot plots for data presented in Fig. 4 E-H and L-P. E’ shows the effect of EP on the expression of PD-L1 in the A549 cells.**

**
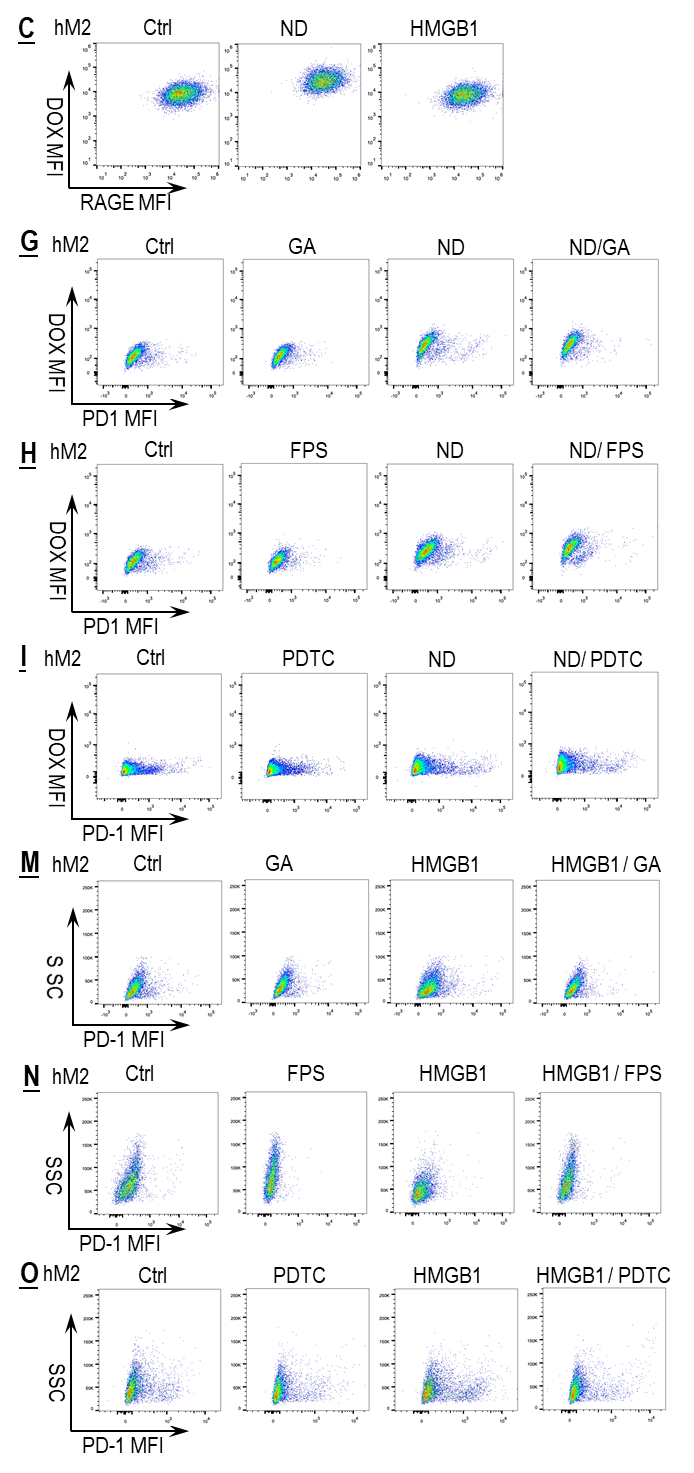
**

**Figure S5. Representative FACS dot plots for data in Fig. 5 C, G-I and M-O.**

**
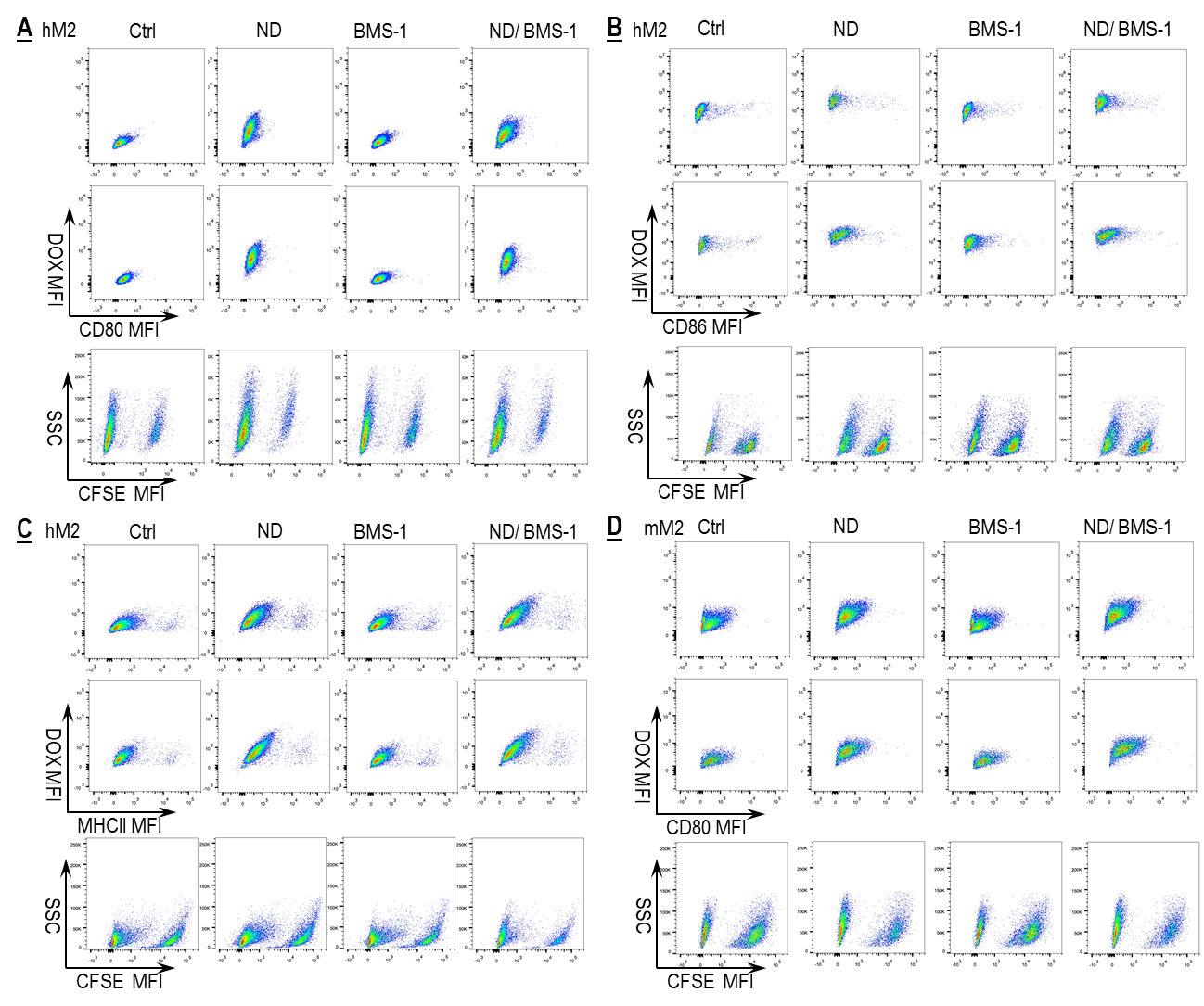
**

**
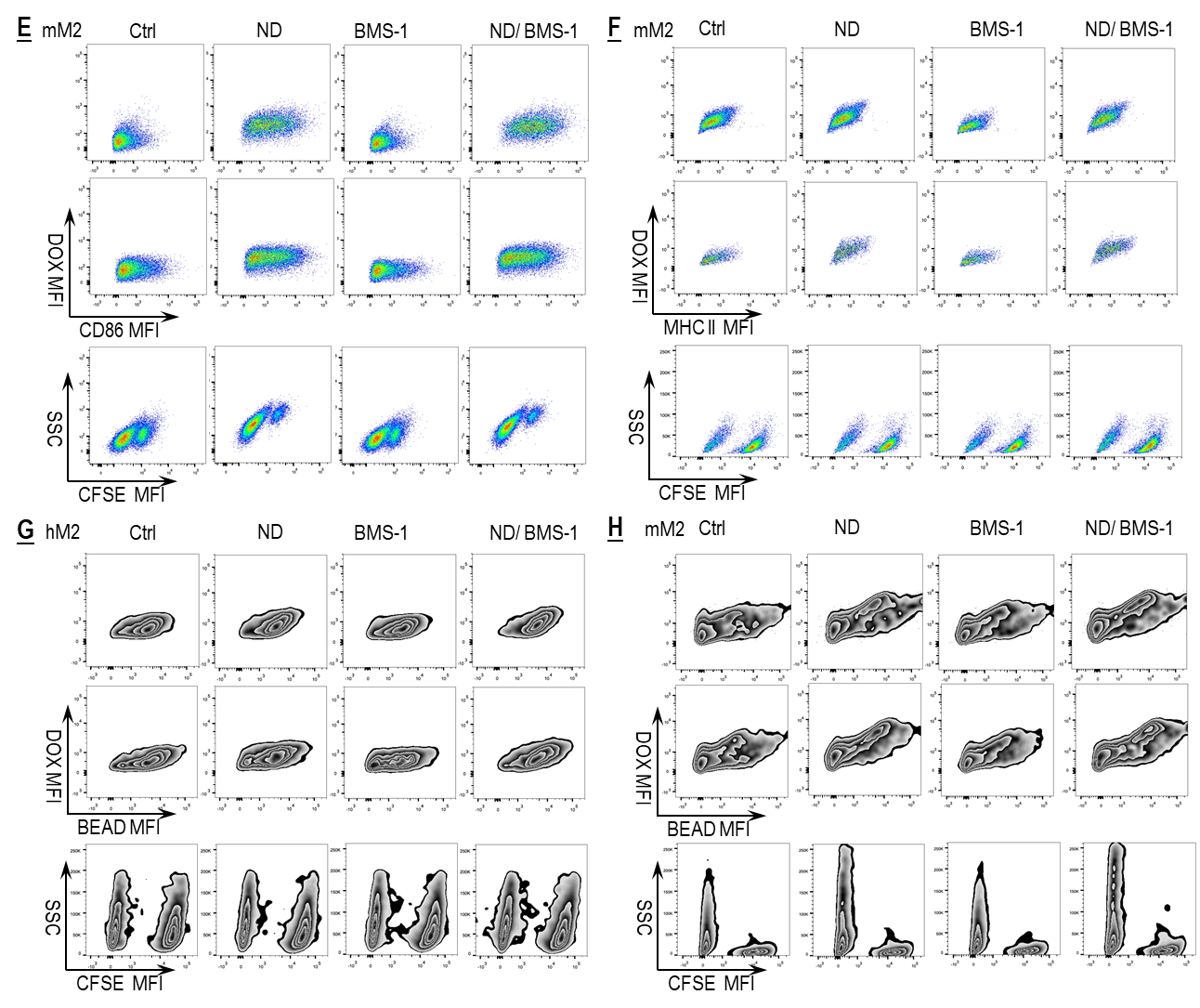
**

**Figure S6 A-H. Representative FACS dot/zebra plots for data in Fig. 6 A-H.**


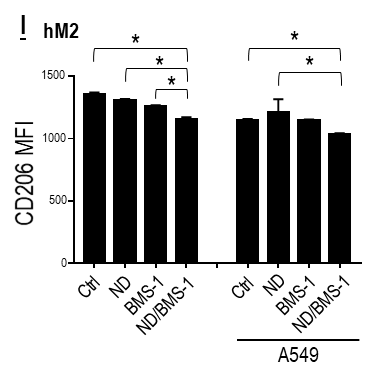

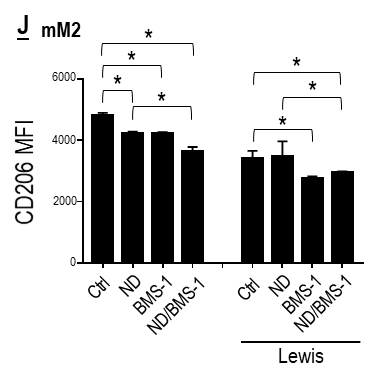


**
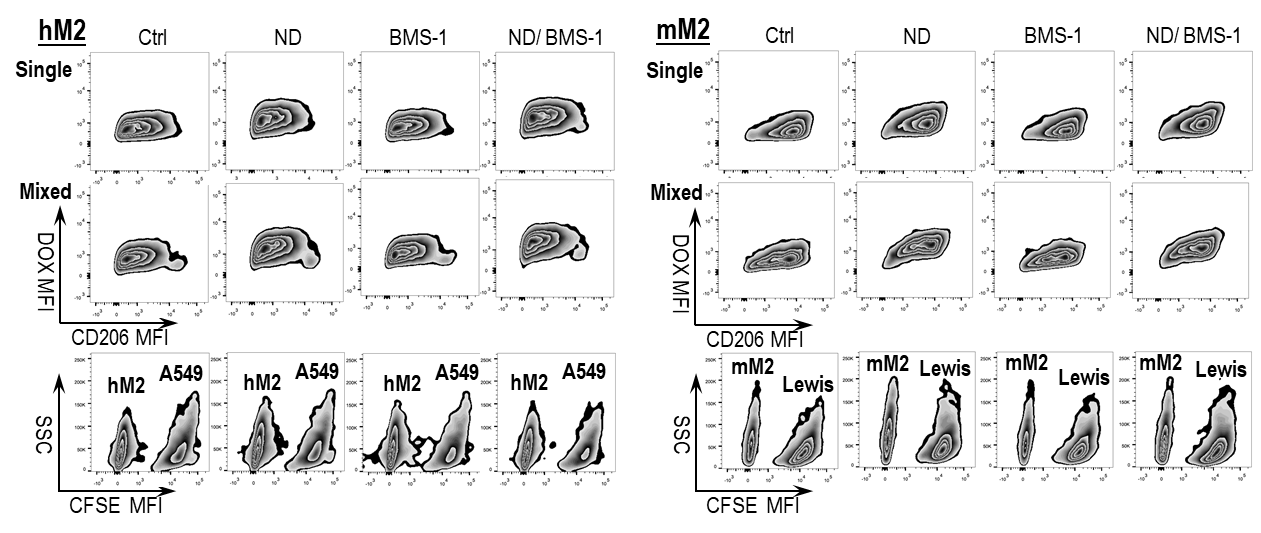
**

**Figure S6 I, J: BMS-1 and Nano-DOX synergistically downregulated surface CD206 in hM2 and mM2.** Cell surface CD206 was assayed by FACS analysis of immunofluorescent staining. FACS histogram geometric means were used to quantify mean fluorescence intensity (MFI). Values were means ± SD (*n = 3, *p < 0.05*). Drug concentration was 2 μg/mL for DOX and Nano-DOX. Treatment duration was 24 hr.

**
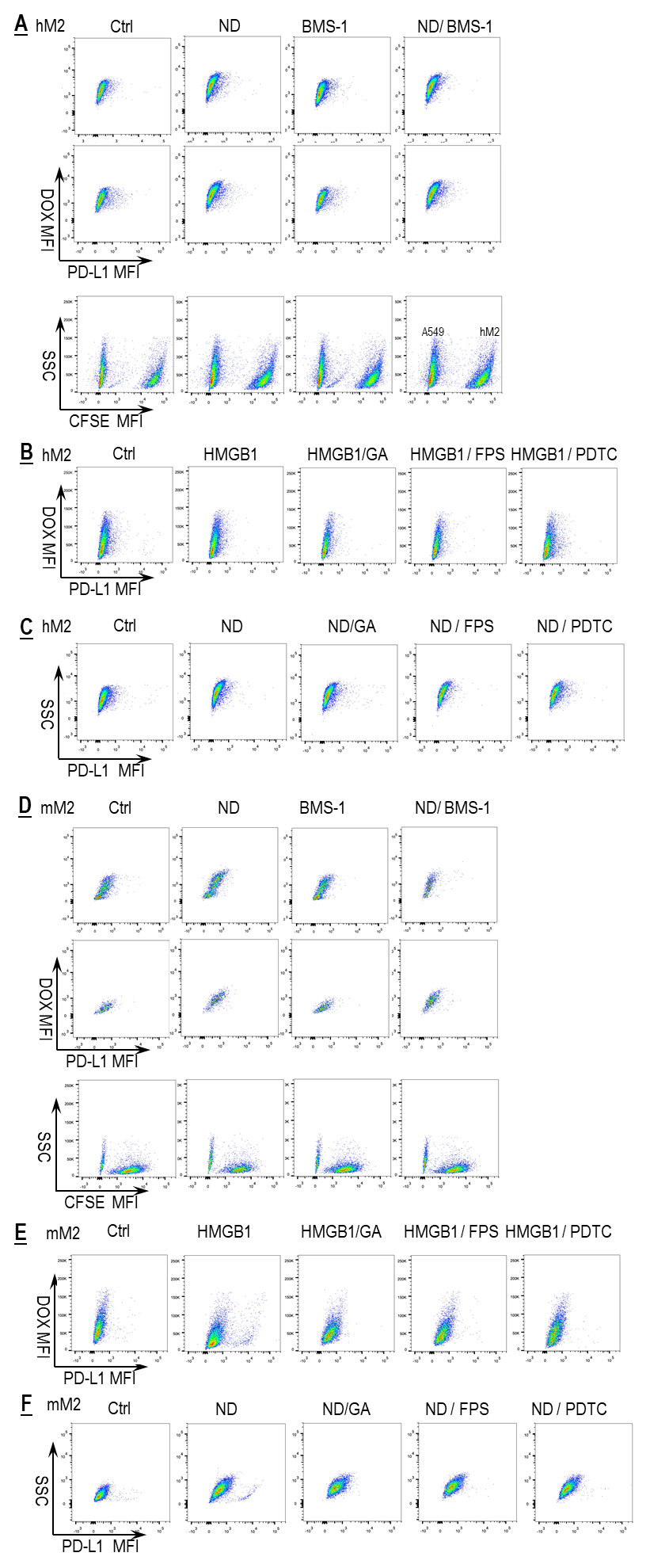
**

**Figure S7. Representative FACS dot plots for data in Fig. 7 A-F.**

**
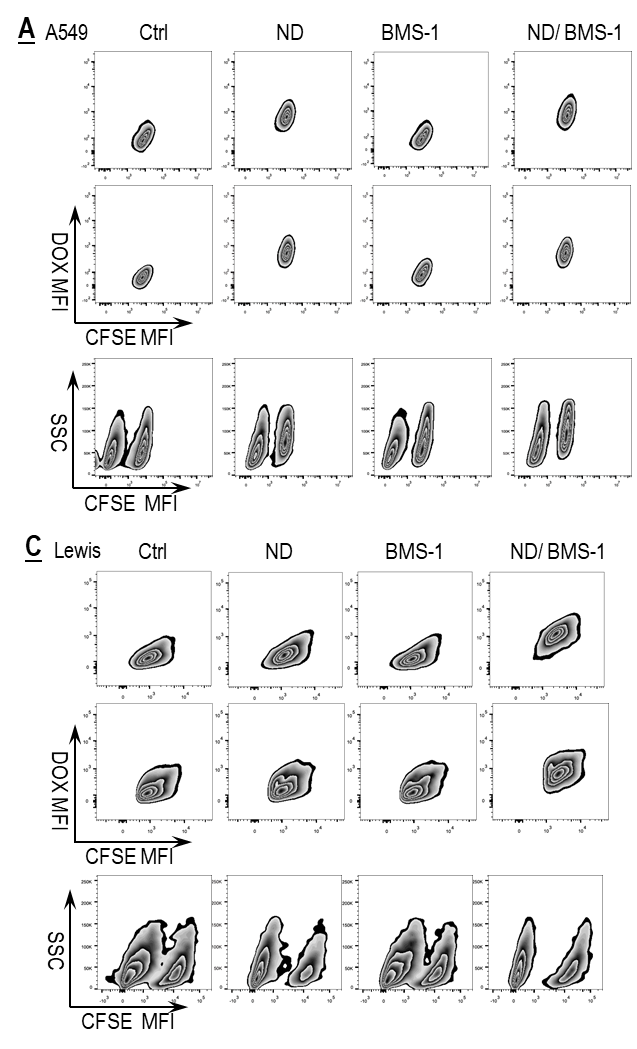
**

**Figure S8. Representative FACS zebra plots for data in Fig. 8 A-D.**

**
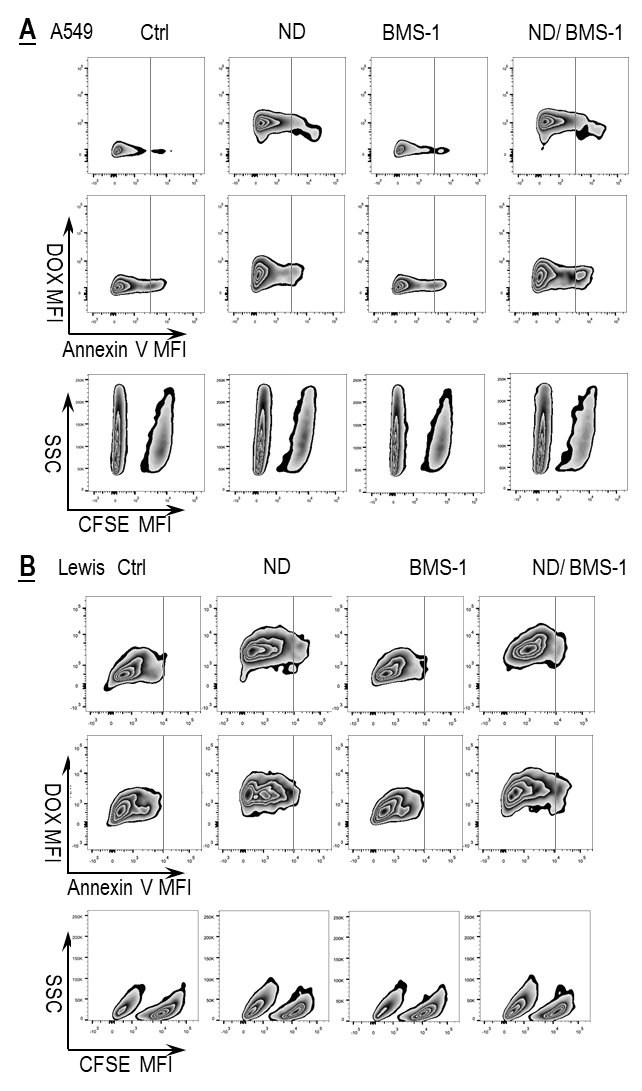
**

**Figure S9. Representative FACS zebra plots for data in Fig. 9 A and B.**

**
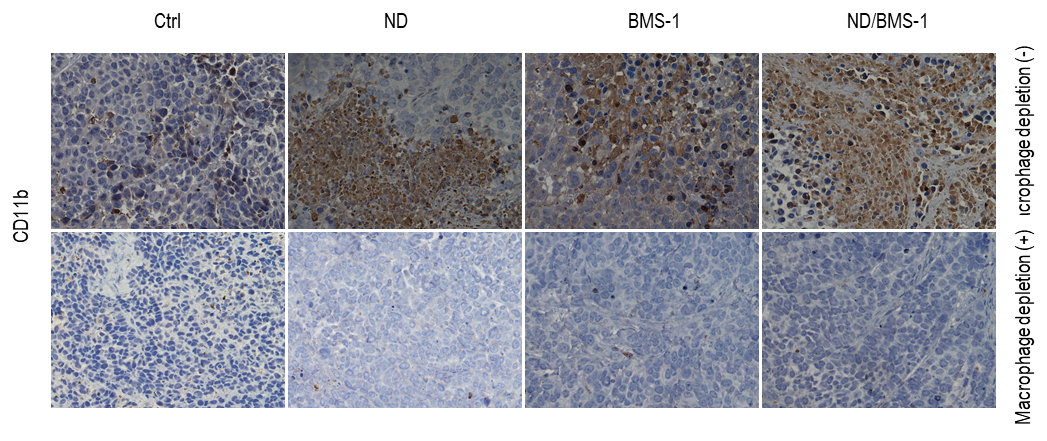
**

**Figure S10. Macrophage depletion in the tumors confirmed by IHC analysis of macrophage surface marker CD11b.**

**
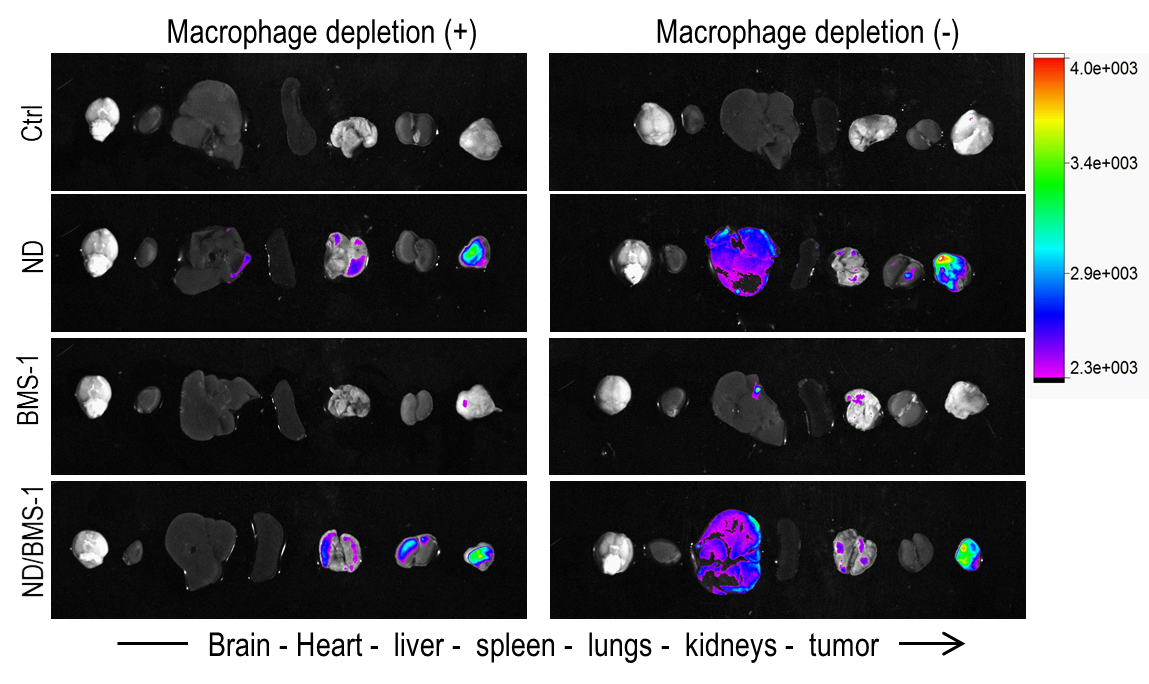
**

**Figure S11. Ex vivo imaging showing drug fluorescence in tumor xenografts and vital organs. ND: Nano-DOX.**

**
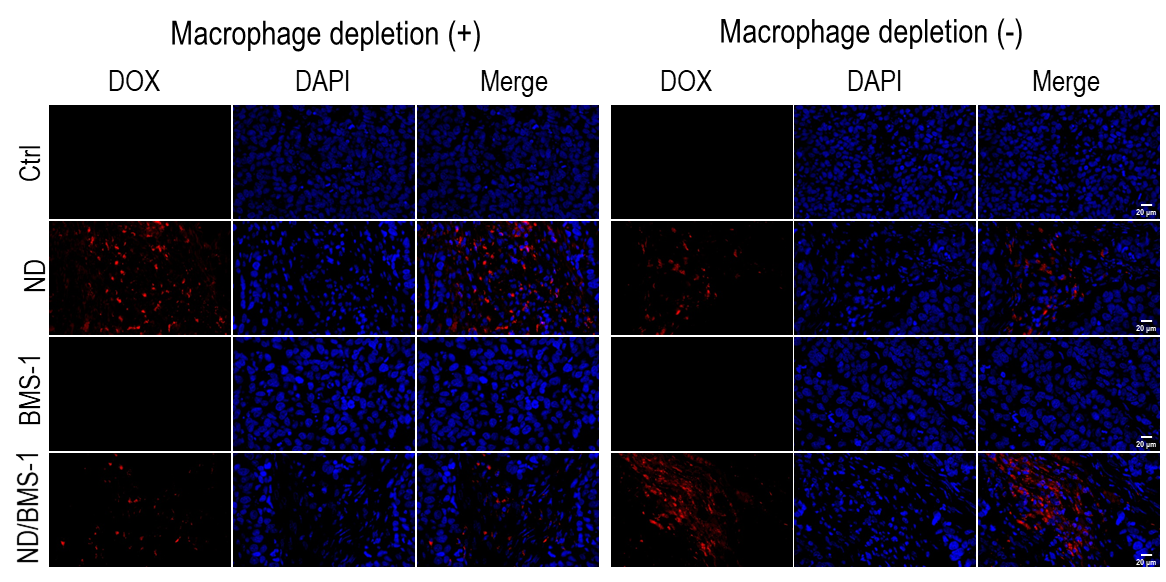
**

**Figure S12. Fluorescent imaging of tumor shows the presence of Nano-DOX. Blue fluorescence is nuclear staining of DAPI. Red fluorescence comes from Nano-DOX (ND).**

**
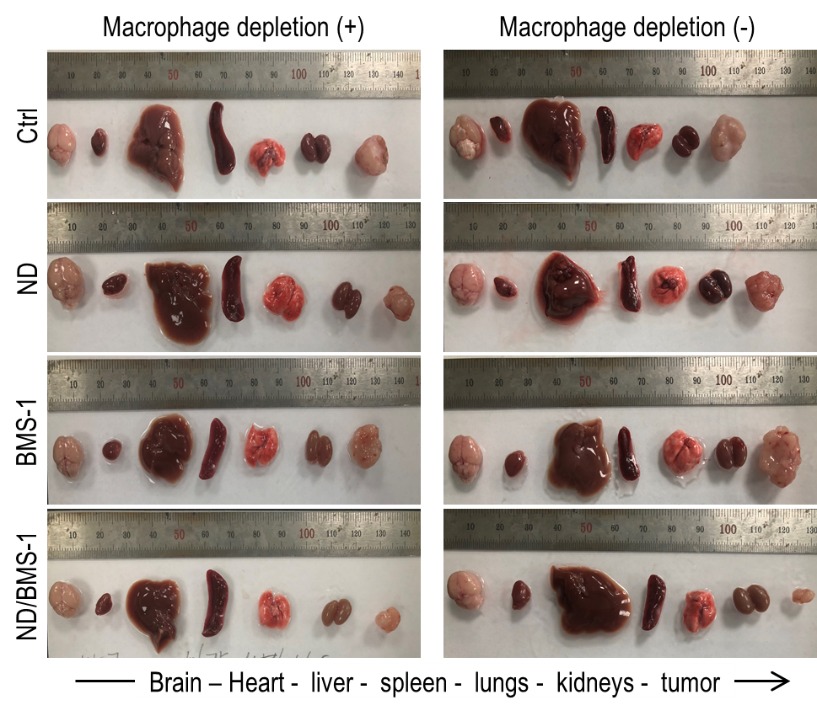
**

**Figure S13. Tumor xenografts and vital organs excised from sacrificed animals.**

**
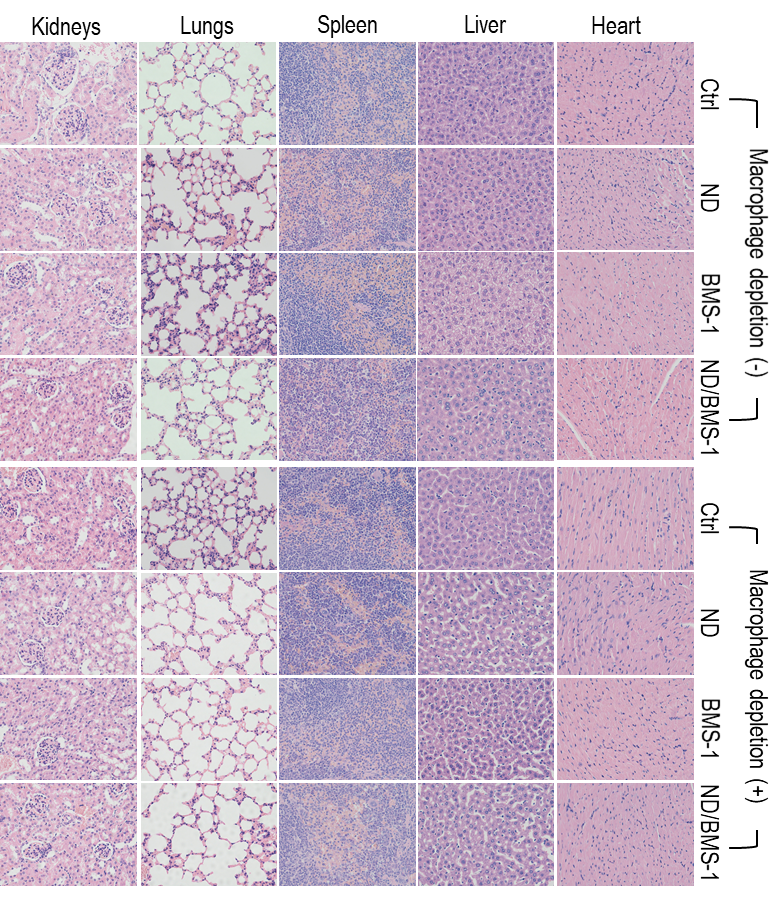
**

**Figure S14. H&E staining of major organs from the mice.**

**
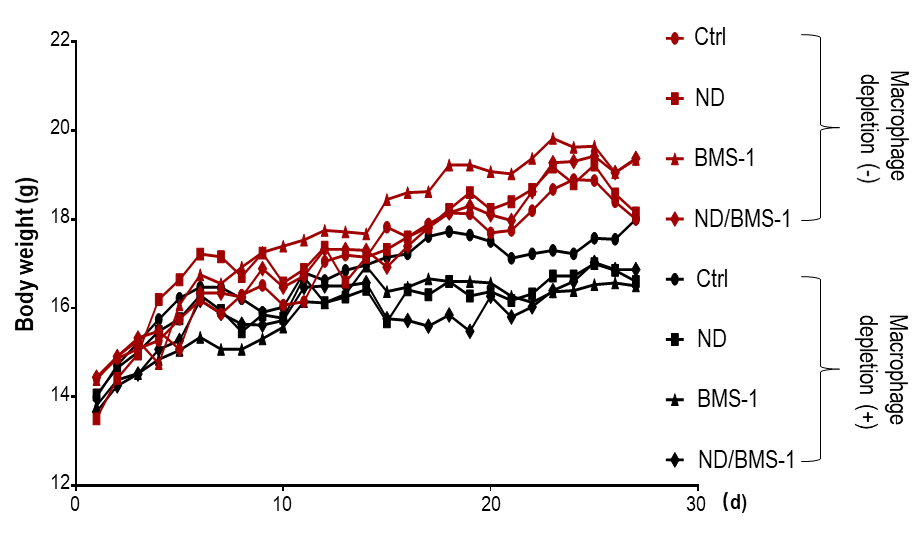
**

**Figure S15. Body weight curves of tumor bearing mice.**
